# Conserved and Differential Effects of Dietary Energy Intake on the Hippocampal Transcriptomes of Females and Males

**DOI:** 10.1371/journal.pone.0002398

**Published:** 2008-06-11

**Authors:** Bronwen Martin, Michele Pearson, Randall Brenneman, Erin Golden, Alex Keselman, Titilola Iyun, Olga D. Carlson, Josephine M. Egan, Kevin G. Becker, William Wood, Vinayakumar Prabhu, Rafael de Cabo, Stuart Maudsley, Mark P. Mattson

**Affiliations:** 1 Laboratory of Neurosciences, National Institute on Aging Intramural Research Program, Baltimore, Maryland, United States of America; 2 Laboratory of Clinical Investigation, National Institute on Aging Intramural Research Program, Baltimore, Maryland, United States of America; 3 Gene Expression and Genomics Unit, National Institute on Aging Intramural Research Program, Baltimore, Maryland, United States of America; 4 Laboratory of Experimental Gerontology, National Institute on Aging Intramural Research Program, Baltimore, Maryland, United States of America; 5 Department of Neuroscience, Johns Hopkins University School of Medicine, Baltimore, Maryland, United States of America; Columbia University, United States of America

## Abstract

The level of dietary energy intake influences metabolism, reproductive function, the development of age-related diseases, and even cognitive behavior. Because males and females typically play different roles in the acquisition and allocation of energy resources, we reasoned that dietary energy intake might differentially affect the brains of males and females at the molecular level. To test this hypothesis, we performed a gene array analysis of the hippocampus in male and female rats that had been maintained for 6 months on either *ad libitum* (control), 20% caloric restriction (CR), 40% CR, intermittent fasting (IF) or high fat/high glucose (HFG) diets. These diets resulted in expected changes in body weight, and circulating levels of glucose, insulin and leptin. However, the CR diets significantly increased the size of the hippocampus of females, but not males. Multiple genes were regulated coherently in response to energy restriction diets in females, but not in males. Functional physiological pathway analyses showed that the 20% CR diet down-regulated genes involved in glycolysis and mitochondrial ATP production in males, whereas these metabolic pathways were up-regulated in females. The 40% CR diet up-regulated genes involved in glycolysis, protein deacetylation, PGC-1α and mTor pathways in both sexes. IF down-regulated many genes in males including those involved in protein degradation and apoptosis, but up-regulated many genes in females including those involved in cellular energy metabolism, cell cycle regulation and protein deacetylation. Genes involved in energy metabolism, oxidative stress responses and cell death were affected by the HFG diet in both males and females. The gender-specific molecular genetic responses of hippocampal cells to variations in dietary energy intake identified in this study may mediate differential behavioral responses of males and females to differences in energy availability.

## Introduction

The energy content and frequency of meals are fundamental aspects of nutrition that can have significant effects on the health of laboratory animals. The reduction of energy intake, or caloric restriction (CR), has been shown to increase both the health span and life span of many species, including rats and mice [1]–[3], fruitflies [4], nematodes [5], water fleas, spiders and fish [2]. On the other hand, consuming excessive food and calories leads to obesity and morbidity and increases the risk of developing type 2 diabetes and cardiovascular disease [6]. Complex neuroendocrine systems control feeding and energy expenditure in mammals; these regulatory systems include cognitive and motivational systems in the brain and hormones produced by endocrine cells and adipose cells [7]–[9]. Many of the physiological effects of reduced energy intake (*e.g*. reduced fat and muscle mass, reduced body temperature, decreased morbidity and mortality) and dietary energy excess (*e.g*. obesity, insulin resistance, cardiovascular disease) are similar in male and female animals. However, there is also evidence that male and female mammals respond differently to alterations in energy intake, both on a neuroendocrine level and on a cognitive level.

In a previous study [10] we demonstrated that a high level of caloric restriction (40% CR) had differential effects upon the physiology and behavior of male and female rats. We demonstrated that there was a beneficial effect of CR (20% CR and 40% CR) upon hippocampus-dependent maze learning in female compared to male rats. Recent findings suggest that higher regions of the brain, including those involved in learning and memory (*e.g*. hippocampus), influence energy balance and physiological and behavioral responses to varying energy availability [11], [12]. Human subjects that are obese have been shown to perform poorer than normal weight subjects in cognitive tasks [13], [14], whereas the learning and memory ability of patients with anorexia nervosa has been shown to be similar or even superior to that of control subjects [15], [16]. Additionally, animal studies have demonstrated a detrimental effect of excessive energy intake on cognitive performance [17]. There is currently a lack of knowledge of the genetic alterations that occur in the hippocampus as a result of alterations in dietary energy intake. It is also unclear whether any potential hippocampal transcriptome changes are differentially regulated in male and female animals. As we have previously demonstrated that there are significant sex-differences in cognitive performance between calorie restricted male and female rats, we sought to determine whether there would be any gender differences in hippocampal gene regulation in response to dietary energy alterations.

In this study, we analyzed the hippocampal gene expression profiles of male and female rats that had been maintained for 6 months on either a control diet, a 20% CR diet, a 40% CR diet, an intermittent fasting (IF) diet or a high fat/high glucose diet (HFG). We identified individual genes, and functional groups of genes that interact with each other in physiological pathways using PAGE (Parametric Analysis of Gene Expression; [18]) which uses a systems biology approach to simulate dynamic changes of specific physiological gene pathways in response to intrinsic processes or external simulation. We found that the hippocampi from the female rats on the low energy diets (20% CR, 40% CR, IF) exhibited conserved changes in gene expression, whereas males did not. Additionally, the CR diets significantly increased hippocampal volume in the female rats, but not in the male rats. Our findings reveal gender-specific molecular genetic responses of hippocampal cells to variations in dietary energy intake, which may determine gender-specific differences in behavioral responses to energy availability.

## Results

### Body weight and hippocampal weight responses to dietary energy restriction and excess

**Figure 1:**
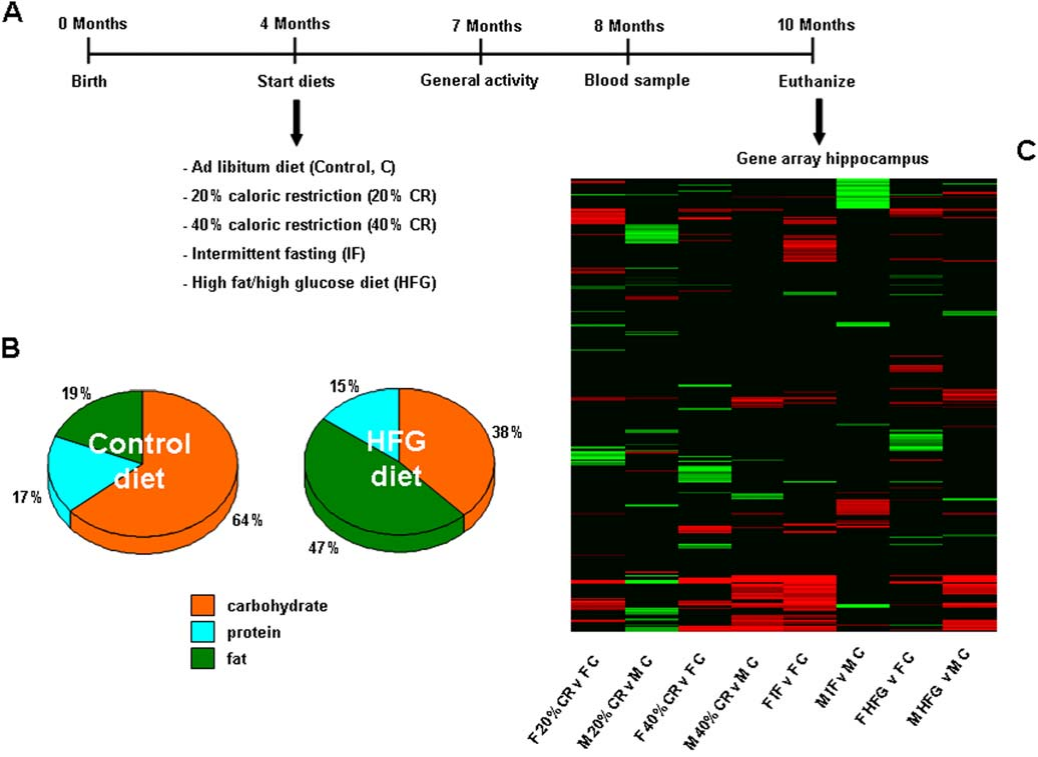
Experimental design, diet composition and a heat map of the significantly altered genes. (A) The experimental timeline for this study. (B) The relative proportions of the major nutritional groups in the control and high-fat/glucose (HFG) diets. (C) A heat map of the significantly up-regulated (red) and down-regulated (green) genes in hippocampi collected from male and female rats on the different dietary regimes, compared to hippocampi collected from *ad libitum* controls.

Four-month-old male and female Sprague Dawley rats were divided into five diet groups: control (*ad libitum*), 20% CR, 40% CR, IF and HFG (8–15 animals per group; Fig. 1A). The rats in the first four diet groups were fed a diet with a typical composition in which the majority of calories were from complex carbohydrates, whereas the HFG diet contained higher amounts of fat and glucose (Fig. 1B). Rats were maintained on the diets for 6 months and when they were 7 months old, general activity was measured and at 8 months of age, a blood sample was collected from each rat. Upon completion of the study, rats were euthanized and the hippocampus was dissected out and collected for transcriptome analyses. The hippocampal gene changes of rats on the different dietary regimes were compared to hippocampal genes from the *ad libitum* control rats and significant gene alterations (up- or down-regulation) were reported. The heat map (Fig. 1C) represents the variety of up- (red) and down- (green) regulated genes that were altered between the diets.

**Figure 2:**
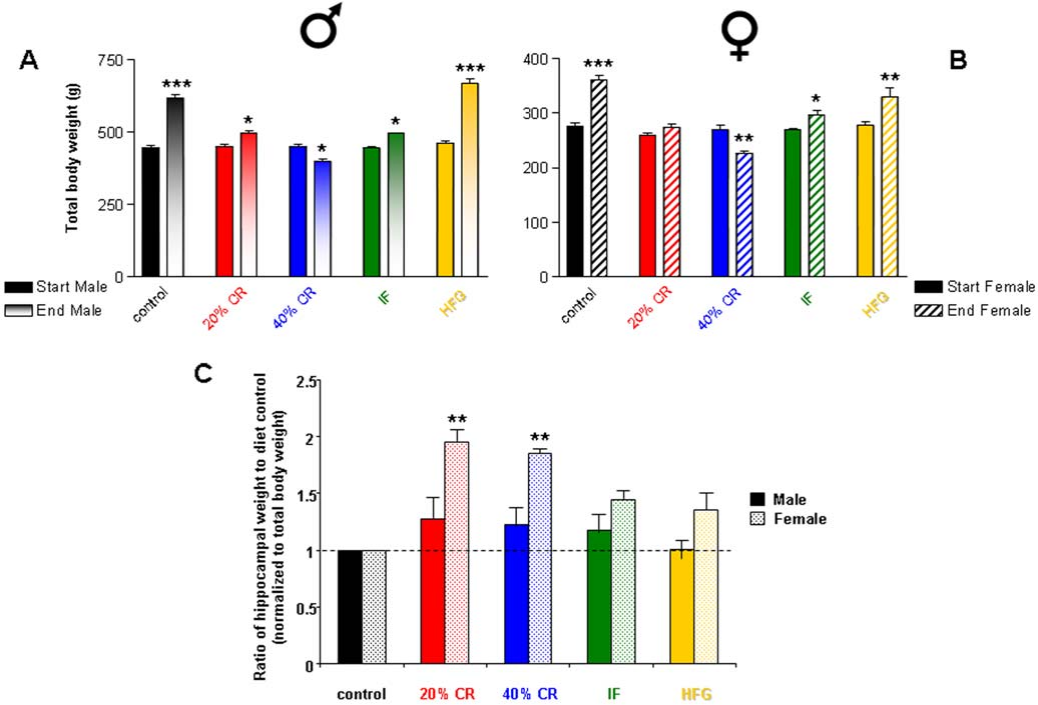
Body and hippocampal weight response to dietary restriction and excess. (A) Male rats on the control, 20% CR, IF and HFG diets had gained a significant amount of body weight at the end of the study. Male rats on the 40% CR dietary regime had lost a significant amount of body weight at the end of the study. (B) Female rats showed a similar response in body weight to the different dietary regimes as the male rats, except for the 20% CR dietary group, which had not gained a significant amount of body weight at the end of the study. (C) The ratio of the hippocampal weights of the rats on 20% CR, 40% CR, IF and HFG diets to the weights of the hippocampi of the rats on the control diet. The male rats showed a small, but non-significant increase in hippocampal weight in response to the three energy restriction diets (20% CR, 40% CR, and IF). The female rats showed increases in hippocampal weight in response to all four dietary manipulations, which were statistically significant for the 20% and 40% CR diets. Values are the mean±SEM for 15 rats in the control diet group and 8 rats in each of the other diet groups. *p<0.05, **p<0.01, ***p<0.001.

Body weight was recorded on a regular basis for each rat [10]. The increase in body weight was significantly greater in the male rats on the control and HFG diets, compared with the increases observed in the female rats (Fig. 2A,B). Whereas males on the HFG diet showed a greater increase in weight than those on the control diet, females on HFG and control diets gained similar amounts of weight. Males and females exhibited similar body weight responses to 20% CR and IF diets (*i.e*. a small increase in body weight). Both male and female rats responded to 40% CR by losing a significant amount of body weight during the study. In addition to measuring total body weight for each rat in the different dietary groups, we measured the weight of the left hippocampus at necropsy and normalized the hippocampal weight to the terminal body weights of rats in the same diet group (Fig. 2C). The male rats on the three energy restricted diets (20% CR, 40% CR, and IF) showed only minimal increases in hippocampal weight compared to *ad libitum* controls. Females on all three of the energy restricted diets showed a considerably greater increase in hippocampal weight than the males (Fig. 2C). This increase in hippocampal mass was statistically significant in the female rats that were subjected to 20% CR or 40% CR. In our previous study [10] we found that 20% CR and 40% CR female rats made significantly less errors in a 14-Unit T maze than *ad libitum* fed control male and female rats. Therefore the female groups with the best maze performance possessed the greatest hippocampal size.

### Dietary energy restriction affects male and female rats similarly with respect to appetite control and glycemia

**Figure 3:**
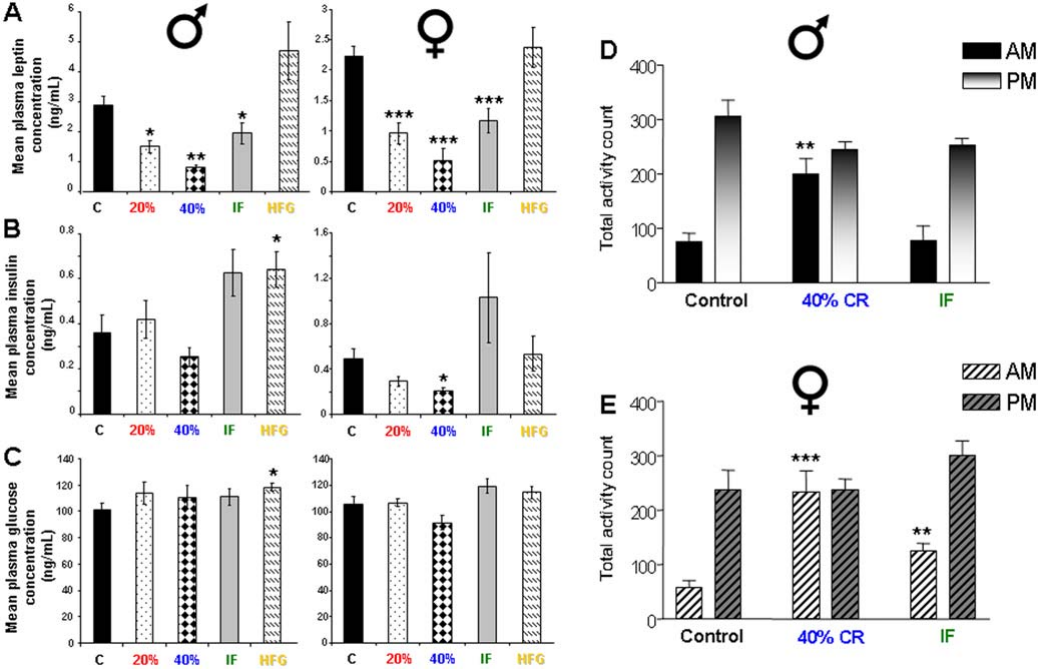
Dietary manipulated male and female rats respond similarly with respect to appetite control, glycemia, and activity. Plasma levels of leptin (A), insulin (B), and glucose (C) were measured in male and female rats at the 4 month on-diet time point. Figures (D) and (E) show the day and night-time activity levels of male and female rats maintained on control, 40% CR, and IF diets. *p<0.05, **p<0.01, ***p<0.001.

As the males and females responded differently to the diets with respect to hippocampal size, we decided to assess whether this discrepancy could be related to differences in appetite perception of the animals. If the males are less sensitive to dietary changes with respect to their appetite, then they may not need to alter their behavior, which then subsequently results in alterations in hippocampal size. Circulating levels of leptin, insulin, and glucose were measured in the male and female rats at the 4 month on-diet time point (Fig. 3A–C). Leptin, the body's satiety hormone, acts as an energy regulator, decreasing appetite and increasing metabolism. Leptin levels in male and female rats were significantly lowered in response to the 20% CR, 40% CR, and IF diets (p<0.001, female 20% CR, 40% CR, and IF; p<0.01, male 40% CR; p<0.05, male 20% CR and HFG). As expected, circulating leptin levels were decreased in response to dietary energy restriction with both the male and female 40% CR groups having the lowest leptin levels compared to *ad libitum* controls. These results suggest that both males and females may have the same hunger perception, and changes in their hippocampus are therefore not due to the males not perceiving less dietary energy intake. Neither males nor females on the HFG diet showed a significant alteration in plasma leptin levels, but there was a trend towards increased leptin levels in both groups (Fig. 3A). Plasma insulin levels tended toward being lower in males and females on the 40% CR diets, although the reduction reached statistical significance only in females on the 40% CR diet (Fig. 3B). Fasting plasma glucose concentrations (Fig. 3C) were similar in males and females and only the male rats on the HFG diet showed a significant increase in glucose levels (p<0.05). Overall, the females and males responded in a similar manner with respect to their appetite and their energy intake, suggesting that additional physiological mechanisms are occurring in females that override their general appetite perception.

### Females increase their ambulatory activity more than males in response to energy restriction

We monitored the total activity of the male and female rats in the control, 40% and IF dietary groups during 4-hour time-periods in the day and night (Fig. 3D, E). The females in the 40% CR group showed a significant increase in daytime total activity, whereas the male rats in the 40% CR group also showed increased total activity (2.3 fold over basal), but to a lesser extent than the 40% CR females (4.2 fold over basal). Additionally, in the females the general activity during the day-time testing period was doubled in response to the IF diet, whereas the IF diet did not affect the general activity levels in the males. Measurements of general activity during the nighttime period showed that general activity during this time period was unaffected by energy restriction in both males and females.

### Significant alterations in the hippocampal gene transcriptome between males and females in response to energy restriction and excess

**Figure 4:**
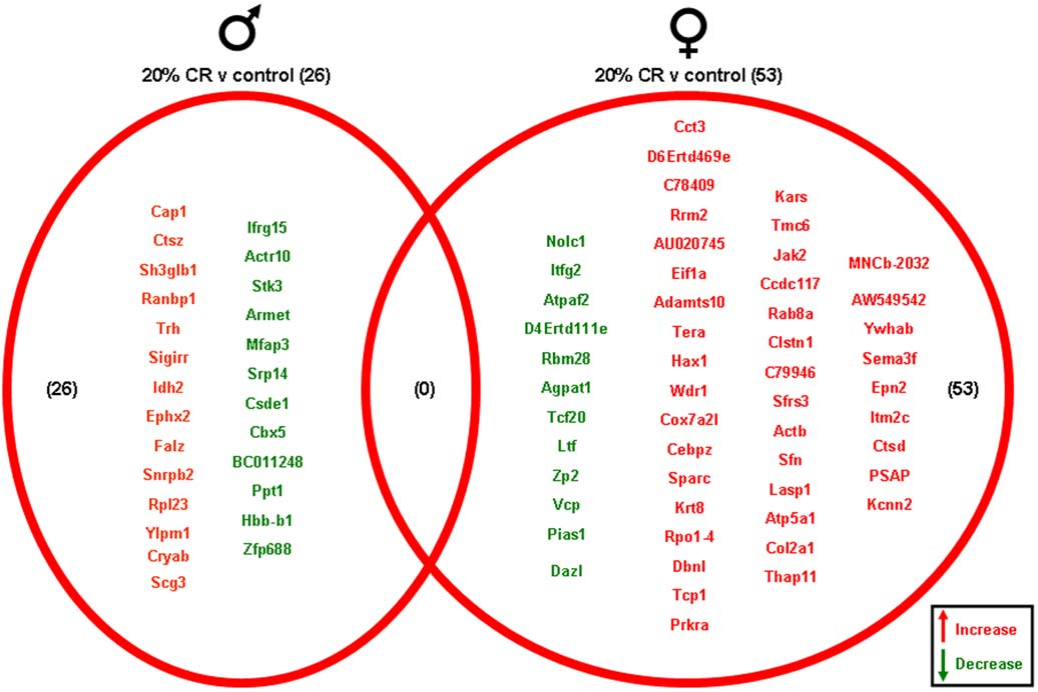
Hippocampi of male and female rats maintained on the 20% CR diet were compared to the hippocampi of male and female rats maintained on a control (*ad libitum*) diet. Genes that were significantly up-regulated (red) or down-regulated (green) were clustered into a Venn diagram. There were 14 significantly up-regulated and 12 significantly down-regulated genes in the hippocampi collected from 20% CR male rats compared to the genes from hippocampi collected from control (*ad libitum*) male rats. Hippocampi collected from 20% CR female rats showed 41 significantly up-regulated (red) and 12 significantly down-regulated (green) genes compared to the hippocampi collected from control (*ad libitum*) female rats. There were no significantly up-regulated or down-regulated common genes between male and female rats in this dietary group. Names of the significantly altered genes can be found in Table S1.

**Figure 5 pone-0002398-g005:**
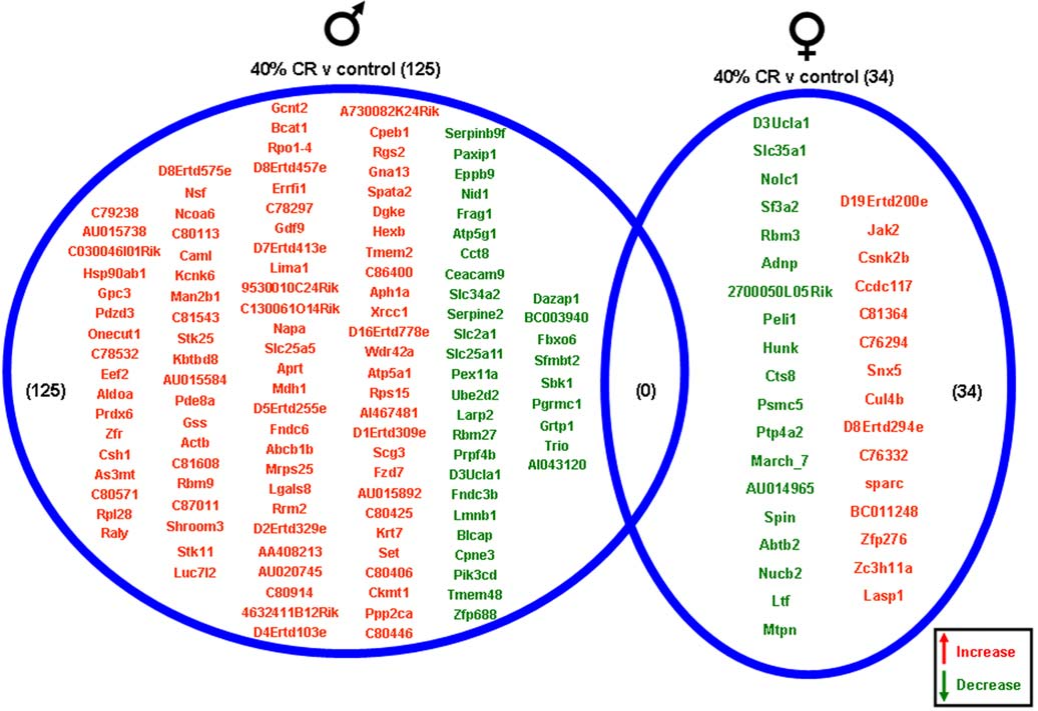
Hippocampi of male and female rats maintained on a 40% CR diet were compared to the hippocampi of rats maintained on a control (*ad libitum*) diet. Genes that were significantly up-regulated (red) or down-regulated (green) were clustered into a Venn diagram. There were 91 significantly up-regulated and 34 significantly down-regulated genes in the hippocampi collected from 40% CR male rats compared to the genes from hippocampi collected from control (*ad libitum*) male rats. Hippocampi collected from 40% CR female rats showed 15 significantly up-regulated (red) and 19 significantly down-regulated (green) genes compared to the hippocampi collected from control (*ad libitum*) female rats. There were no significantly up-regulated or down-regulated common genes between male and female rats in this dietary group. Names of the significantly altered genes can be found in Table S1.

**Figure 6 pone-0002398-g006:**
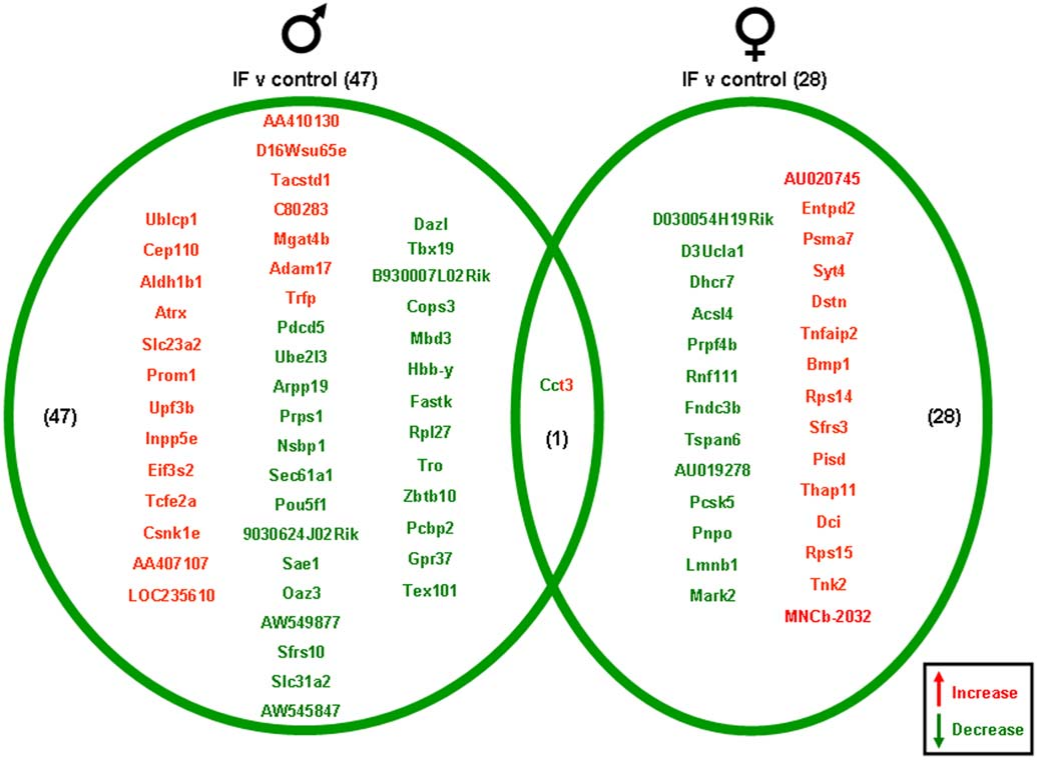
Hippocampi of male and female rats maintained on an IF diet were compared to the hippocampi of rats maintained on a control (*ad libitum*) diet. Genes that were significantly up-regulated (red) or down-regulated (green) were clustered into a Venn diagram. There were 20 significantly up-regulated and 27 significantly down-regulated genes in the hippocampi collected from IF male rats compared to the genes from hippocampi collected from control (*ad libitum*) male rats. Hippocampi collected from IF female rats showed 15 significantly up-regulated (red) and 13 significantly down-regulated (green) genes compared to the hippocampi collected from control (*ad libitum*) female rats. There was one gene that was significantly altered in both the males and females in the IF dietary group compared to the control group. This gene, Cct3, was significantly down-regulated in the IF males and was significantly up-regulated in the IF females, compared to control (*ad libitum*) males and females. Names of the significantly altered genes can be found in Table S1.

**Figure 7:**
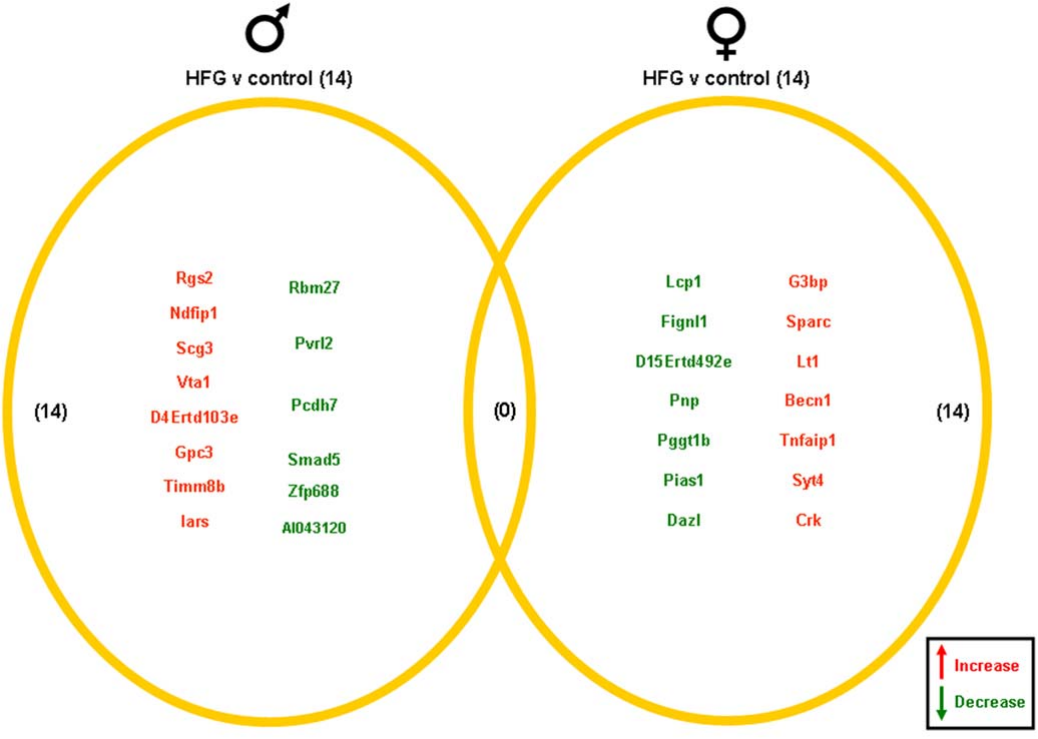
Hippocampi of male and female rats maintained on a HFG diet were compared to the hippocampi of rats maintained on a control (*ad libitum*) diet. Genes that were significantly up-regulated (red) or down-regulated (green) were clustered into a Venn diagram. There were 8 significantly up-regulated and 6 significantly down-regulated genes in the hippocampi collected from HFG male rats compared to the genes from hippocampi collected from control (*ad libitum*) male rats. Hippocampi collected from HFG female rats showed 7 significantly up-regulated (red) and 7 significantly down-regulated (green) genes compared to the hippocampi collected from control (*ad libitum*) female rats. There were no significantly up-regulated or down-regulated common genes between male and female rats in this dietary group. Names of the significantly altered genes can be found in Table S1.

Significantly altered genes in the hippocampi of male and female rats on the various dietary regimes compared to genes from hippocampi from the *ad libitum* controls are summarized in the Venn diagrams (Fig. 4–7, Table S1). The 20% CR males had 26 significant hippocampal gene alterations, compared to *ad libitum* male controls. Of these significantly altered genes, 14 were up-regulated and 12 were down-regulated. The 20% CR female rats had 53 significantly altered hippocampal genes compared to controls, more than double the number of significantly altered hippocampal genes in the male 20% CR rats (Fig. 4). In the 20% CR females, 41 of the significantly altered genes were up-regulated and 12 were down-regulated. The 40% CR males had 125 hippocampal genes that were significantly altered compared to male controls. Of these significantly altered genes, 91 were up-regulated and 34 were down-regulated. Interestingly, the gene alterations in the hippocampi of the 40% CR male rats was 3 times more than the gene alterations in the hippocampi of 40% CR females. In the hippocampi of the 40% CR females, there were 34 genes that were significantly altered; 15 genes were significantly up-regulated and 19 genes were significantly down-regulated (Fig. 5). The IF males had 47 significant hippocampal gene alterations compared to the male controls, and 20 of these genes were up-regulated and 27 were down-regulated. The IF females had a total of 28 significantly altered hippocampal genes, and 15 of these genes were up-regulated and 13 were down-regulated. Additionally, there was one gene, Cct3 (Chaperonin containing T-complex polypeptide 1, subunit 3), that was altered in the hippocampi of both males and females on the IF diet. This gene was significantly down-regulated in the male hippocampi and significantly up-regulated in the female hippocampi (Fig. 6). Cct3 is a chaperonin involved in the proper folding of the cytoskeleton proteins actin and tubulin [19]. Males and females on the HFG diet each had 14 significant hippocampal gene alterations compared to controls. The male HFG rats had 8 significantly up-regulated genes and 6 significantly down-regulated genes. Females on the HFG diet had 7 significantly up-regulated genes and 7 significantly down-regulated genes (Fig. 7).

### Female hippocampal gene regulation is calorie specific

**Figure 8:**
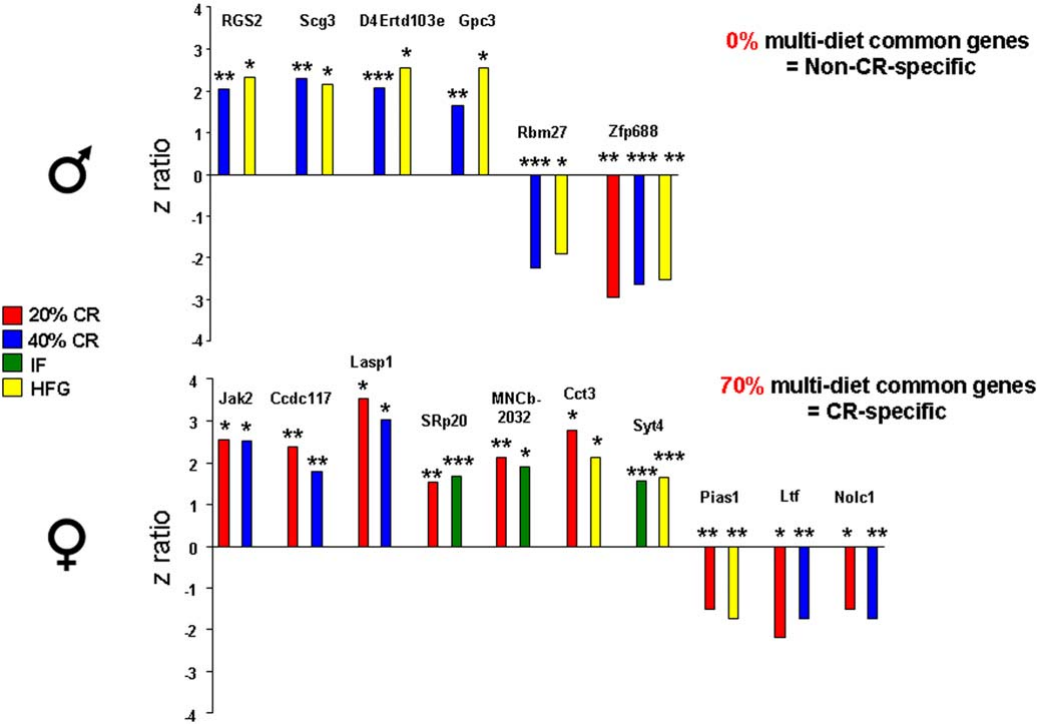
Female hippocampal gene regulation is energy intake-specific. The significantly up-regulated or down-regulated genes that were common between the different dietary regimes. Male hippocampal gene regulation was found to be conserved between the very low energy diet (40% CR) and the high energy (HFG) diet, and interestingly there were no genes in the males that were common between the different energy restriction diets (20% CR, 40% CR, IF). Female hippocampal gene regulation was found to be significantly conserved (70%) between all the energy restriction diets (20% CR, 40% CR, IF), suggesting that female gene regulation is energy intake-specific. Unlike the males, there were no significantly conserved genes between the very low energy diet (40% CR) and the high energy diet (HFG) in the females. *p<0.05, **p<0.01, ***p<0.001.

Genes that were common and conserved (altered in the same direction) between at least two different diets are reported in Fig. 8. The males showed 100% commonality between genes modified coherently between the 40% CR and HFG diets. These two diets represent the greatest difference of intaken energy. While these two diets are calorically very different, they are probably the two most stressful dietary regimes; therefore, it is especially interesting that the genetic response to these two conditions were so similar in the males. The females, on the other hand, showed 70% coherency between the energy restriction diets (20% CR, 40% CR, and IF), indicating that their genetic response to dietary manipulation was very specific and calorie dependent. The organization of the females' response to the energy restricted diets is suggestive of some underlying mechanism that may allow for an organized, pre-programmed, response to enhance survival in times of food scarcity. Comparatively, the males' genetic response was less specific, suggesting that the males respond to a general stressor but they seem to lack the ability to discriminate between a high energy and low energy stressor.

### Hippocampal functional gene pathways are differentially altered in males and females in response to dietary energy restriction and excess

**Figure 9:**
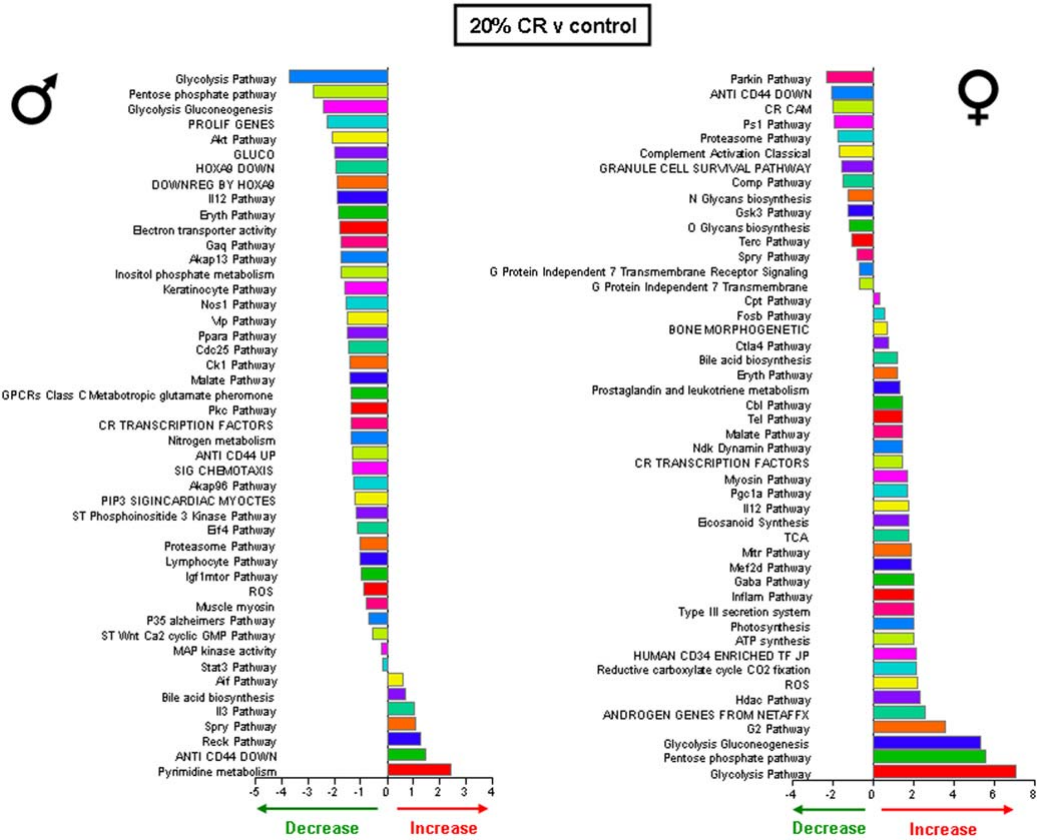
Significant gene pathway changes in the hippocampi of male and female rats maintained on a 20% CR diet. Significantly altered genes in the male and female hippocampi from the different dietary regimes were clustered into functional gene pathways. In the hippocampi from male rats on the 20% CR diet, there were 47 significantly altered gene pathways, of which 40 pathways were significantly down-regulated and 7 pathways were significantly up-regulated, compared to gene pathways in hippocampi from male control rats. Interestingly, the hippocampi from female rats on the 20% CR diet showed a very different functional gene pathway pattern as there were 48 significantly altered pathways, of which 15 were significantly down-regulated and 33 were significantly up-regulated, compared to gene pathways in hippocampi from control female rats.

**Figure 10 pone-0002398-g010:**
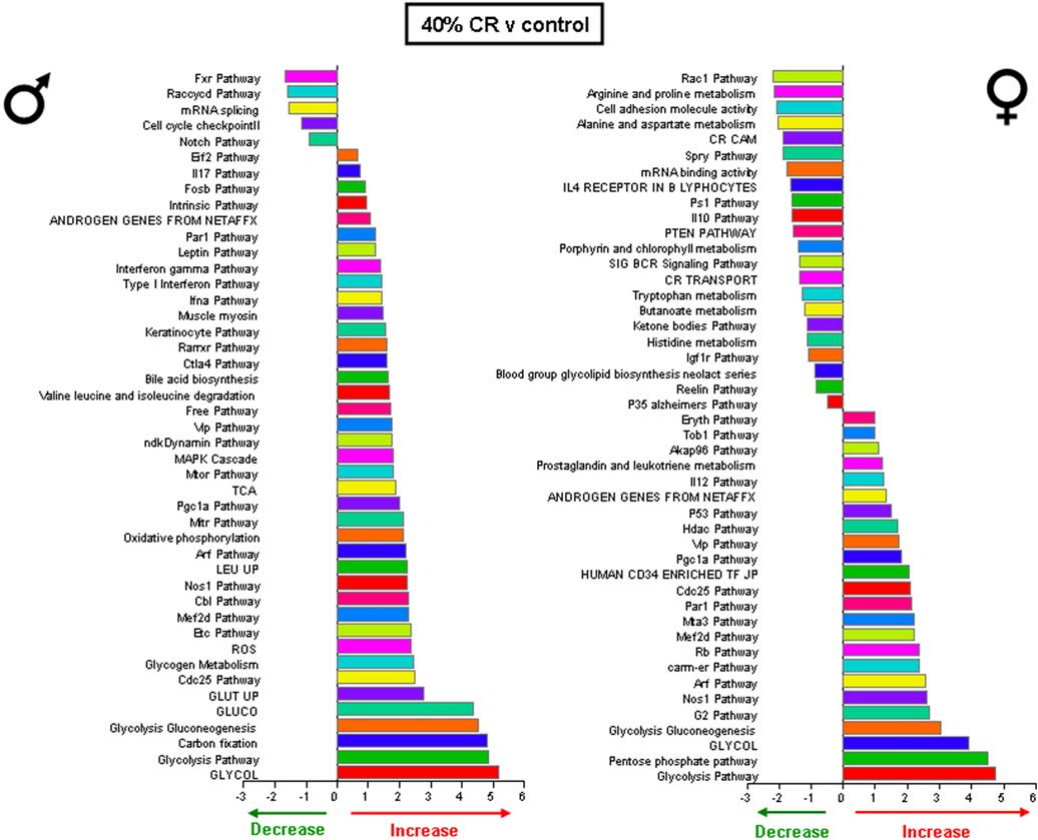
Significant gene pathway changes in the hippocampi of male and female rats maintained on a 40% CR diet. Significantly altered genes in the male and female hippocampi from the different dietary regimes were clustered into functional gene pathways. In the hippocampi from male rats on the 40% CR diet, there were 45 significantly altered gene pathways, of which 5 pathways were significantly down-regulated and 40 pathways were significantly up-regulated, compared to gene pathways in hippocampi from male control rats. Interestingly, the hippocampi from female rats on the 40% CR diet showed a very different functional gene pathway pattern as there were 46 significantly altered pathways, of which 22 were significantly down-regulated and 24 were significantly up-regulated, compared to gene pathways in hippocampi from control female rats.

**Figure 11 pone-0002398-g011:**
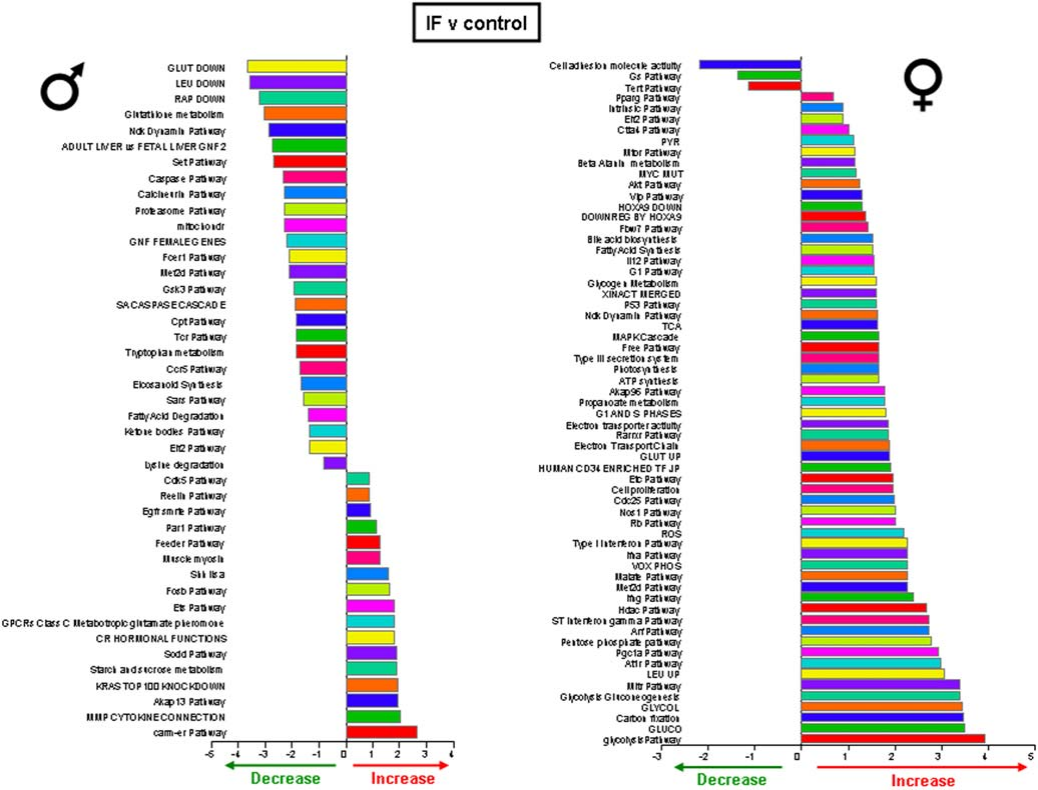
Significant gene pathway changes in the hippocampi of male and female rats maintained on the IF diet. Significantly altered genes in the male and female hippocampi from the different dietary regimes were clustered into functional gene pathways. In the hippocampi from male rats on the IF diet, there were 43 significantly altered gene pathways, of which 26 pathways were significantly down-regulated and 17 pathways were significantly up-regulated, compared to gene pathways in hippocampi from male control rats. Interestingly, the hippocampi from female rats on the IF diet showed a very different functional gene pathway pattern as there were 63 significantly altered pathways, of which 3 were significantly down-regulated and 60 were significantly up-regulated, compared to gene pathways in hippocampi from control female rats.

**Figure 12:**
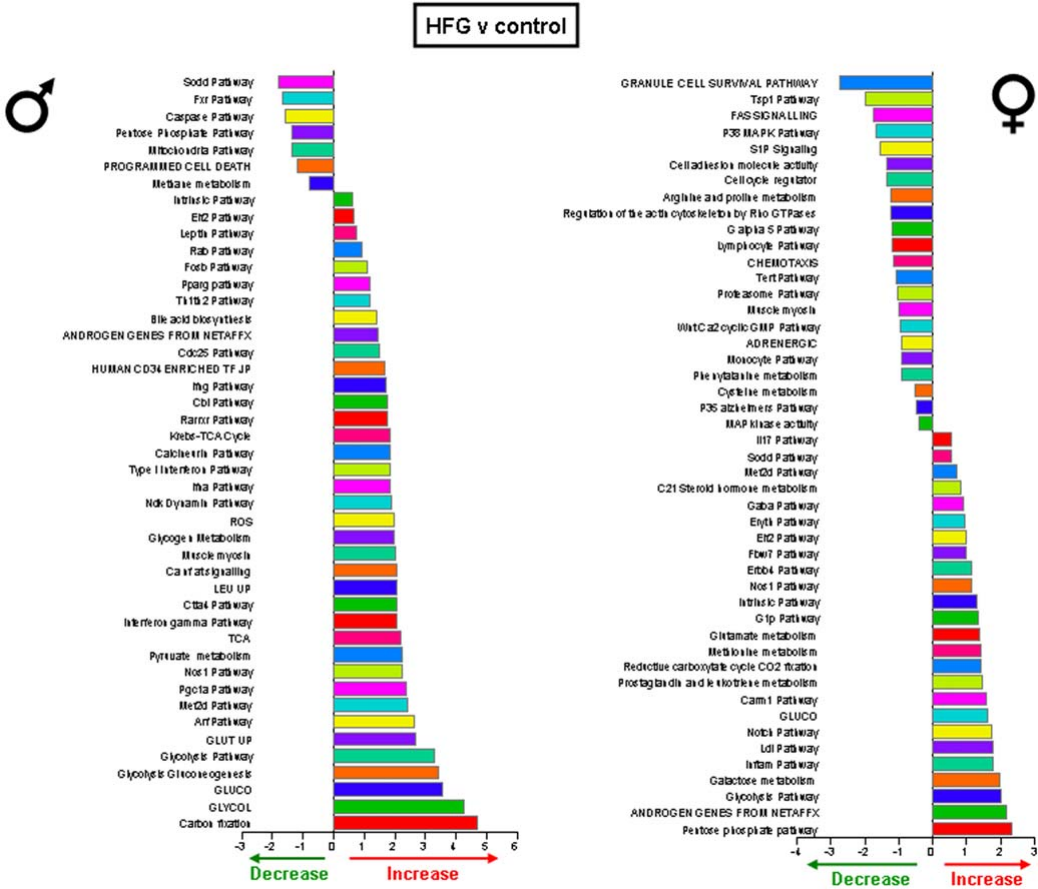
Significant gene pathway changes in the hippocampi of male and female rats maintained on a HFG diet. Significantly altered genes in the male and female hippocampi from the different dietary regimes were clustered into functional gene pathways. In the hippocampi from male rats on the HFG diet, there were 45 significantly altered gene pathways, of which 7 pathways were significantly down-regulated and 38 pathways were significantly up-regulated, compared to gene pathways in hippocampi from male control rats. Interestingly, the hippocampi from female rats on the HFG diet showed a very different functional gene pathway pattern as there were 47 significantly altered pathways, of which 22 were significantly down-regulated and 25 were significantly up-regulated, compared to gene pathways in hippocampi from control female rats.

Significantly altered hippocampal genes were grouped into functional categories, established by the Broad Research Institute at MIT, to form 522 functional gene pathways. The up- or down- regulation of these functional gene pathways in the hippocampi of the rats on the various dietary regimes (compared to *ad libitum* fed controls) was analyzed and is summarized in Fig. 9–12. The male rats on the 20% CR diet showed 47 altered gene pathways, 40 of these pathways were significantly decreased, and 7 were significantly increased (Fig. 9). Interestingly, while the female rats on the 20% CR diet showed a similar number of significantly altered pathways (48) the direction of the majority of pathway alterations was opposite to the pathways of the males. In the 20% CR females, 15 pathways were significantly decreased, and 33 pathways were significantly increased (Fig. 9). The males on the 40% CR diet showed 45 significantly altered pathways. The majority of these altered pathways were increased (40), while only 5 were decreased (Fig. 10). The female rats on the 40% CR diet had a fairly equal number of up- and down-regulated pathways. Of 46 significantly altered pathways, 22 were decreased and 24 were increased (Fig. 10). The males on the IF diet showed 43 significantly altered hippocampal pathways, and 26 of these were decreased and 17 were increased (Fig. 11). Out of a total of 63 significantly altered pathways in the hippocampi of IF females, only 3 were down-regulated, and the remaining 60 were up-regulated (Fig. 11). Males on the HFG diet had 45 significantly altered hippocampal pathways; 7 were decreased and 38 were increased (Fig. 12). Females on the HFG diet had a fairly even number of up- and down-regulated pathways. Out of 47 significantly altered pathways, 22 were decreased and 25 were increased (Fig 12).

In males, the 20% CR diet down-regulated pathways were pathways involved in glycolysis and mitochondrial ATP production, whereas these metabolic pathways were up-regulated in females (Fig. 9). Additional pathways involved in cellular energy metabolism and nutrient sensing that were up-regulated by 20% CR in females included protein deacetylation and PGC1-α. The proteasome pathway was down-regulated by 20% CR in both males and females. The 40% CR diet up-regulated pathways were pathways involved in glycolysis, protein deacetylation, PGC-1α and mTor pathways in both sexes (Fig. 10). IF down-regulated many gene pathways in males including those involved in protein degradation and apoptosis, but up-regulated many gene pathways in females including those involved in cellular energy metabolism (glycolysis, gluconeogenesis, pentose phosphate pathway, electron transport and PGC1-α), cell cycle regulation and protein deacetylation (Fig. 11). Gene pathways involved in energy metabolism, oxidative stress responses and cell death were affected by the HFG diet in both males and females (Fig. 12). Further descriptions of specific genes and pathways of interest that were affected by diets and gender are included in the Discussion section below.

### Female hippocampal gene pathway regulation is calorie specific

**Figure 13:**
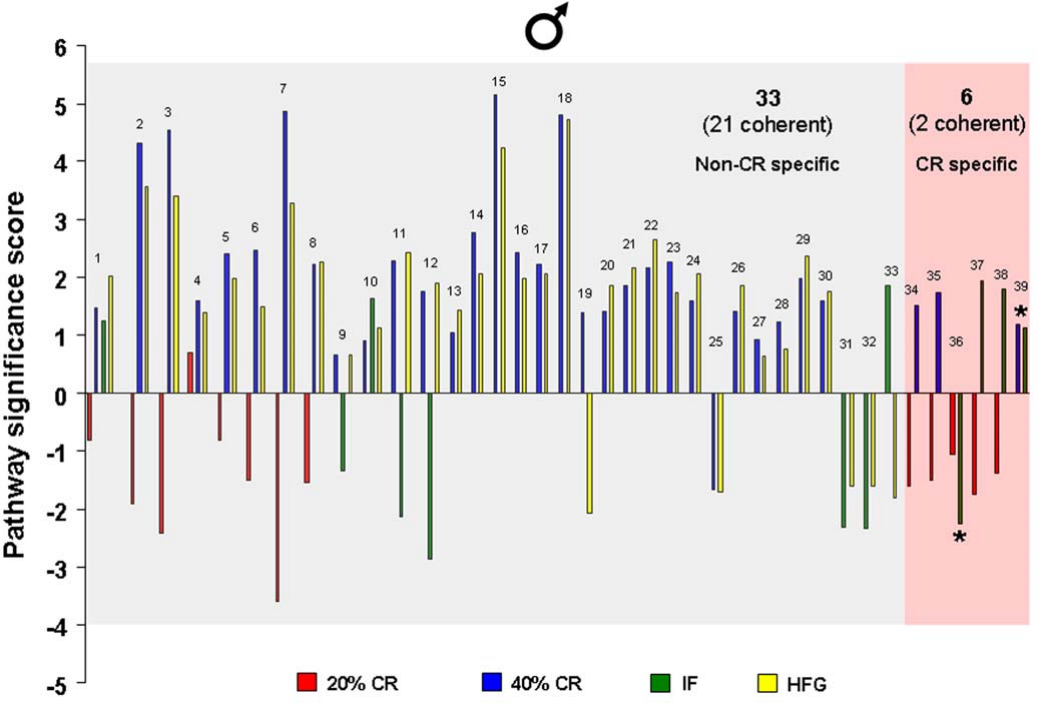
Males respond in an energy intake independent manner to dietary restriction and excess. The common-diet, significantly altered pathways in male hippocampi are summarized. There were similar changes in gene pathways between the 40% CR and HFG dietary groups, which suggests that the male hippocampal gene response to dietary restriction and excess was not calorie specific. Names of the significantly altered pathways can be found in Table S2.

**Figure 14 pone-0002398-g014:**
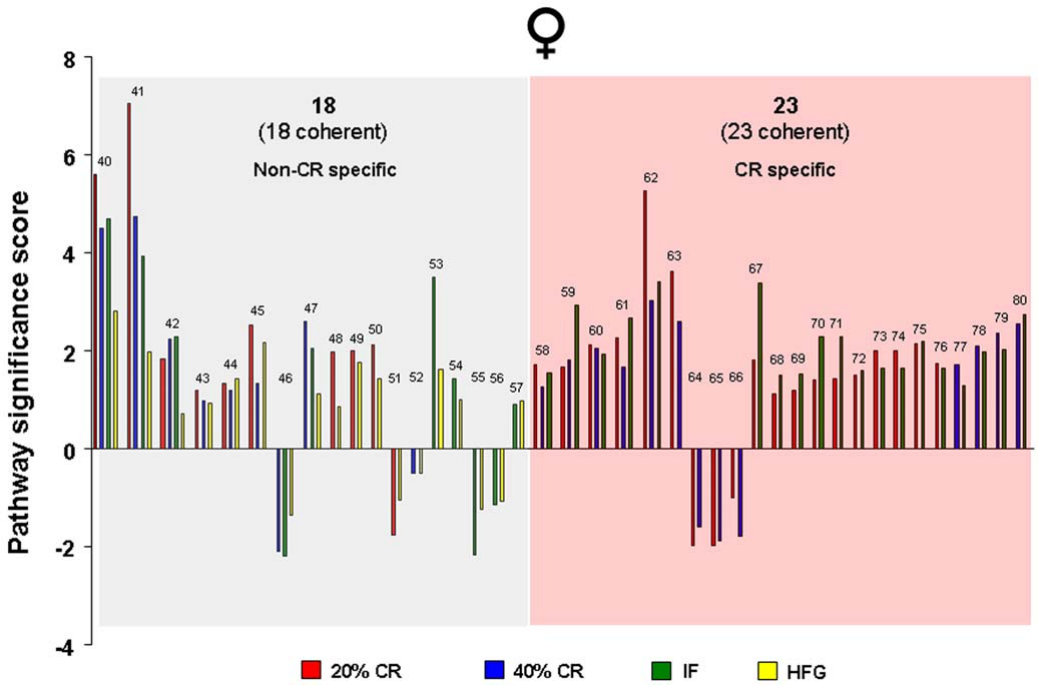
Females respond to dietary restriction and excess in an energy intake-dependent manner. The common-diet, significantly altered pathways in female hippocampi are summarized. More than half of the significantly altered gene pathways were common and coherent (altered in the same direction) between the energy restriction diets (20% CR, 40% CR, and IF), which suggests that the female hippocampal gene response to dietary manipulation is CR-dependent. Names of the significantly altered pathways can be found in Table S2.

Specific gene patterns within the common and conserved hippocampal gene pathways were analyzed further to determine the number of pathways that were common between the low energy and high energy dietary groups (Fig. 13–16, Table S2). In response to the dietary alterations, the male rats showed a significant commonality in genetic pathway response between the 40% CR and HFG diets (Fig. 13); 33 out of a total of 39 significantly altered hippocampal pathways were common between the HFG diet and at least one of the reduced energy diets (20% CR, 40% CR, or IF). Of these common pathways, 21 were coherent, meaning that they were altered in the same direction (either up- or down-regulated) between the diets. However, only 6 pathways in the male hippocampi were affected by one or more of the reduced energy diets. Further underlining the lack of a coherent genetic response in the males to energy intake, is that only 2 of the 6 pathways affected by energy restriction were regulated in a coherent manner. It is interesting to note that in complete contrast to the males, the female genetic pathway response was largely dependent upon the level of energy intake. In the females, 23 of a total of 41 significantly altered hippocampal gene pathways were energy restriction-specific. It was striking that for the 23 CR-dependent pathways, and the 18 CR-independent pathways, 100% of the pathways were coherent between diets, meaning that common, significantly altered pathways in the female hippocampi were always altered in the same direction (Fig. 14). This would suggest that, compared to the genetic pathway response of male hippocampi, female hippocampal gene pathway responses are highly organized, especially with respect to caloric restriction. This specific energy-dependent gene pathway organizational response could possibly stem from an evolutionary mechanism to allow females to be well equipped to adequately respond to times of dietary energy scarcity (or excess), so that they could remain fertile and have adequate energy stores to rear their offspring.

**Figure 15 pone-0002398-g015:**
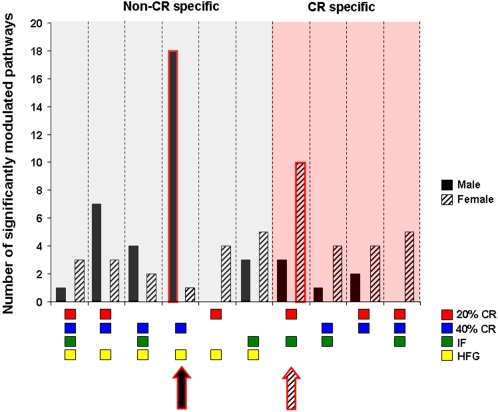
Significantly modulated pathways in the hippocampi of male and female rats in response to dietary restriction and excess. The number of pathways whose alteration overlaps between the different diets is reported. Male rats showed the largest amount of overlap between the 40% CR and HFG pathways, which suggests that their genetic response to dietary manipulation is not CR or energy specific. Female rats on the other hand, showed a significant amount of overlap between the 20% CR and IF diets, which suggests that there is a CR-dependency in genetic response to dietary manipulation in the hippocampi of female rats.

**Figure 16:**
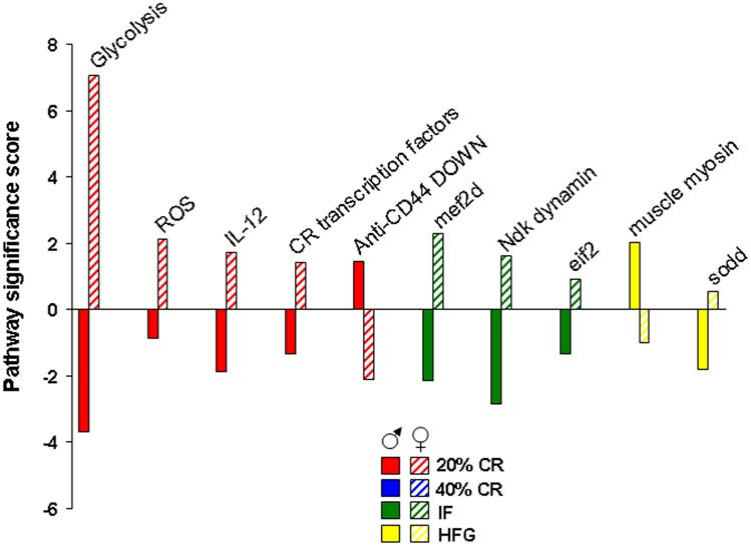
Divergent male/female hippocampal gene pathway responses. Hippocampal gene pathways that were significantly and differentially altered between the hippocampi of male and female rats maintained on the same diet are summarized.

### Specificity of common pathway regulation

Cluster analysis of the low energy diets only, and any low energy diet with HFG common pathways, revealed that certain dietary group combinations showed greater numbers of modulated pathways. As shown in Fig. 15 we have grouped the number of significantly modulated pathways (modulated significantly in at least two or more different dietary paradigms) for all the dietary combinations in which pathway commonalities were seen. Therefore Fig. 15 represents the multi-dietary group combinations that showed either the greatest or least coherency of physiological pathway regulation. It is important to note that for males the non-CR-specific dietary regime combination with the greatest number of coherently regulated pathways was the 40% CR-HFG cluster (*i.e*. highest dietary stress conditions). The female dietary combinations that showed the greatest degree of pathway coherency were the two diets that possess the closest parity in actual input calories, *i.e*. 20% CR and IF [10]. This is an important finding considering the functional differences between these two dietary paradigms. It seems therefore that female hippocampal gene regulation is considerably more sensitive to reduced energy intake compared to males.

To directly contrast the gender differences in hippocampal gene responses to energy restriction we have summarized the hippocampal gene pathways that were differentially regulated in the males and females on the same diets (Fig. 16). The divergent responses of the hippocampal transcriptome in response to identical diets provides a further indication of the gender differences in ‘pre-programmed’ hippocampal gene responses to energy restriction or excess. Some of the prominent functional groups of differentially regulated genes in males and females code for proteins that are involved in energy metabolism and glycolysis, dendritic function and regulation, and anti-apoptotic mechanisms. In the females on 20% CR the major functional gene pathway that was significantly up-regulated was the glycolysis pathway. Interestingly, this same pathway was down-regulated in the males on 20% CR, reinforcing a strong gender bias of hippocampal regulation in response to reduced energy availability.

## Discussion

In the present manuscript, we have demonstrated that there are gender-dependent alterations in hippocampal gene regulation in response to perturbations in dietary energy intake. Hippocampal gene regulation in the female rats was highly conserved between all the energy restricted diets (20% CR, 40% CR, IF), and 70% of multi-diet common genes that were significantly altered (up-regulated or down-regulated) were energy deprivation-specific. In the males, we observed a different phenomenon, as hippocampal gene regulation was not conserved among the energy restricted diets, and none of the multi-diet common genes that were significantly altered were energy deprivation-specific. Interestingly, in the male rats, the only common multi-diet genes that were significantly altered were between the lowest energy (40% CR) and the highest energy (HFG) diet regimes. This suggests that female hippocampal gene regulation is calorie-specific and is very sensitive to varying degrees of dietary energy intake. Male hippocampal gene regulation on the other hand, was not calorie-specific as there were no multi-diet common genes that were energy level-specific. However, as there was a 100% coherence between the lowest energy diet (40% CR) and the highest energy diet (HFG), *i.e.* the two most extreme diets, this could suggest that in the males there was a coherent stress response. This phenomenon in males could potentially be elucidated in future studies by exposing the male and female animals to additional stressors that could alter the performance in females more than males and change cognitive performance and or/strategies on other special tasks, such as the Morris Water Maze.

The functional gene pathway analyses (PAGE) demonstrated that, in the females, there were 23 significantly altered coherent pathways that were energy deprivation-specific and 18 significantly altered coherent gene pathways that were non-specific. Females responded most coherently to mild caloric reductions (20% CR and IF diets). Interestingly, in the male hippocampi, we observed an entirely opposite phenomenon to the female hippocampi. The hippocampi from the male rats on the low energy diets (20% CR, 40% CR and IF) showed no conserved gene alterations, as 100% of the multi-diet common genes were not specific for reduced energy intake. However, it is interesting to note that there were common gene alterations between the very low energy diet (40% CR) and the very high energy (HFG) diet in the male rats. The functional gene pathway analyses performed on the male hippocampi showed that only 6 significantly altered coherent gene pathways were energy deprivation-specific and 21 significantly altered coherent gene pathways were not specific for reduced energy intake. Therefore not only at the gene level, but also at the pathway level, there is a similarity in response of the animals, in that female rats have a selective and largely energy intake-specific genetic/pathway response while the male rats only show commonalities in their genetic/pathway response when extreme dietary perturbations are introduced. These genetic responses therefore may serve to enhance cognitive function and facilitate the females' ability to find and secure food. Additionally, it is possible that the coherence in energy restriction for females could also benefit males from an evolutionary perspective. One could argue that the coherence in energy restriction for females could also benefit males from an evolutionary perspective, thus leaving the impression of a dysfunctional pattern in males possibly related to an exaggerated stress response.

Total activity levels were measured when the rats were 7 months old in the *ad libitum* control, 40% and IF diet groups. The female rats on the 40% CR diet significantly increased their total daytime activity, more so than the 40% CR male rats. This increased ambulatory activity and heightened cognitive ability [10] that we have demonstrated in female rats on a 40% CR diet is consistent with a scenario in which food scarcity imposes a stress on the animal, motivating them to seek food elsewhere. This may be particularly important in female mammals, in contrast to males, because they must ensure that they obtain a sufficient amount of energy to not only support their own survival and fecundity but also the survival and development of their offspring. Diverting any available energy to neuromuscular and cognitive activity would be expected to increase the probability of survival of these females during times when food is scarce. Energy regulating hormones and appetite hormones were, as expected, significantly altered in both the male and female calorie restricted animals.

Circulating levels of leptin were significantly reduced in all rats on reduced energy diets, and the females on the 40% CR diet showed decreased levels of insulin and glucose. This effect is a well-established effect of caloric restriction, which is known to cause decreases in circulating glucose levels and increased insulin sensitivity. It is interesting to note therefore that there may be an evolutionarily-conserved mechanism by which females specifically change their behavior and cognitive capacity in relation to available food. The presence of this heightened sensitivity to low energy intake in females may partly be responsible for the higher prevalence of anorexia nervosa (AN) in females compared to males [20]. Thus when food intake is restricted, either through the environment or voluntarily, only the female will induce a coherent genetic response that affects not only whole body physiology [10] but also higher brain functions such as cognition. The increases in hippocampal size we documented in female rats on CR diets also correlate to changes seen in humans that are involved in task learning [21] or even exercise [22]. These two interventions have been shown to increase hippocampal volume and neurogenesis through the creation of mild, tolerable stressors on the brain, and therefore it is likely that in the female mild CR results in a similar functional phenotype.

With respect to specific genes coherently altered by various dietary paradigms, there are several striking functional interactions, *e.g*. between two genes that conversely regulate cytokine signaling. Hence the 20% CR females show a robust elevation in Jak2 (Janus kinase 2) expression while there is a corresponding reduction in the levels of Pias1 (protein inhibitor of activated STAT). Jak2 tyrosine kinase serves as a primary functional mediator of growth hormone, prolactin and interleukin signaling. Upon ligand binding the receptor, Jak2 is recruited to proline-rich domains in the intracellular regions of the receptor where it activates and then tyrosine phosphorylates the STAT (signal transducers and activators of transcription) families of transcription factors. Pias1 can act to functionally antagonize the generation of active STAT molecules [23]. Pias1 belongs to a family of proteins that promote the conjugation of sumo1 (small ubiquitin-related modifier-1) to different classes of proteins. This post-translational modification of proteins is described as sumoylation [24]. Sumoylation is mechanistically but not functionally related to ubiquitination. Ubiquitination destines target proteins to internalization and eventual degradation. Sumoylation engenders much more diverse actions such as promoting transport from the cytoplasm to the nucleus [25], protection from ubiquitination [26] and regulation of protein-protein interactions [27]. The up-regulation of Jak2 with the down-regulation of Pias1 may mediate effects of dietary energy intake on electrical excitability/gene transcription in the hippocampus. For example, GH can enhance the excitability of hippocampal CA1 neurons [28] in a Jak2 and PI3-kinase-dependent manner, while Pias1 has been shown to directly sumoylate multiple members of the mGluR family of glutamate receptors [25]. We previously reported that there are considerable differences in the levels of GH in males and females in response to caloric restriction, *i.e*. females show increased GH levels whereas males show dramatic diminution of GH levels [10]. Whether this hormone variation alone mediates the cognitive changes seen in females on reduced energy diets is not known, but is consistent with many reports linking maintenance of GH levels in the elderly and preservation of cognitive capacity [for review see 29]. The genes that are coherently controlled by reduced energy diets in females may cooperate to affect functional changes in neuronal networks. For example, several genes control synaptic actin dynamics including Lasp1 and Cct3 [19], [30], synaptic vesicle and protein trafficking (Syt4; [31]) as well as control hippocampal memory formation patterns (SRp20; [32]).

Changes in many different common and cell type-specific genes in the CNS are known to occur in response to many different environmental factors including exercise, age, diet, activity in neuronal circuits, and injury or disease [33]–[39]. Despite the fact that there are multiple phenotypic differences between male and female animals, the vast majority of diet studies and gene expression analyses have been performed only on males, and direct comparisons of hippocampal transcriptome responses of males and females to environmental factors, such as dietary intake, are largely lacking. A recent study has shown that numerous genes, spanning numerous functional categories, were differentially expressed in the CNS of males and females [40]. In general, genes involved in protein degradation, oxidative stress resistance and cell survival were expressed at higher levels in females compared to males, which could suggest a superior ability of brain cells in females to resist oxidative and metabolic stress. Interestingly, the authors of this study found that there was a considerable amount of variability in the number of genes affected by sex among the different regions of the CNS, with the transcriptome of the hippocampus being the most sensitive to sex-differences in response to the one dietary alteration they tested, and the striatum and cerebellum being the least sensitive [40]. This could potentially explain why, in the calorically restricted females, 14-Unit T-maze learning ability was significantly increased [10], since the hippocampus seems to be very sensitive to dietary energy input information. It is also interesting to note that in the males subjected to 40% CR, the total number of regulated genes was substantially greater than at 20% CR, this is perhaps indicative of a greater genetic response needed to maintain energy homeostasis in the face of severe caloric restriction. In contrast, the 40% CR females that demonstrated the greatest learning improvement in the 14-Unit T-maze in our previous study displayed a much smaller range of genetic modulation, perhaps suggesting the presence of a pre-programmed capacity, or ‘*genetic engram*’, to change in response to the caloric restriction. In future studies, we plan to relate the present findings and prior behavioral results of dietary manipulations to other cognitive tasks (e.g. the Morris Water Maze) where females typically show a performance decrement compared to males. Also, it would be very informative to detail changes in other brain structures that contribute to learning and memory besides the hippocampus.

It will be important to gain a deeper understanding of the molecular mechanisms that underpin these sex-dependent alterations that occur in hippocampal transcriptomes in response to different levels of dietary energy intake. Gaining a greater appreciation of how male and female hippocampal cells respond to different levels of caloric intake could illuminate some of the potential downstream signaling pathways that are altered in a sex-dependent manner. The proteins and further cell signalling events down-stream of the genetic hippocampal transcriptome that are differentially regulated in males and females could determine sex-specific differences in responses to dietary energy intake and potentially even neurocognitive behaviors and general susceptibility to disease.

## Materials and Methods

### Animals and diets

47 male and 47 female Sprague-Dawley rats were singly housed on a 12 hr light/dark cycle. The following diets were applied to the rats beginning at 4 months of age: control (*ad libitum*); 20% CR, 40% CR; IF (alternate day fasting); and HFG. Control, CR and IF groups received food pellets that contained 19% protein, 64% carbohydrates, and 17% fat (diet 101845 from Dyets Inc., Bethlehem, PA); this food had a caloric density of 3.774 cal/g and a glycemic load/kg of 442. The HFG diet (diet 101842 from Dyets Inc.) contained 15% protein, 38% carbohydrates, and 47% fat. The caloric density of the HFG diet was 4.645 cal/g and its glycemic load/kg was 363. Weights were recorded for each rat on a regular basis throughout the study. All procedures were performed in accordance with approved institutional protocols and were approved by the Institutional Animal Care and Use Committee of the National Institute on Aging.

### Assessment of ambulatory activity

Total activity levels were quantitated using an Omnitech Digiscan open-field activity monitor (Columbus, Ohio, USA) when the rats were 7 months old. Recordings were taken in 4 h intervals (from 7:00 to 10:00 and again from 19.00 until 22.00). Additionally, throughout the study activity levels were also observed and scored by multiple observers at random times during the day. Using the latter semi-quantitative scoring system of general activity, it became apparent that only the 40% CR animals and IF animals showed increased daytime activity. The rat activity levels were therefore formally measured (using the Omnitech apparatus) at one time-point during this study, as our observations showed that animal activity levels did not change throughout the study.

### Tissue and plasma collection

At 8 months of age, overnight-fasted rats were sedated with isoflurane and blood samples were collected from the tail vein to EDTA-heparinized centrifuge tubes. The blood was centrifuged at 3,000 rpm for 30 min at 4°C; plasma was aspirated and was stored at −80°C. At the end of the study, the rats were euthanized using isoflurane anaesthesia followed by decapitation. Upon euthanasia, the brain was carefully dissected to obtain the hippocampus. Tissues were flash frozen on dry ice and stored at −80°C until further analyses.

### ELISA analyses

Plasma levels of the following hormones were measured according to the manufacturers' instructions using proprietary ELISA kits from the specified companies: leptin (ELISA-Linco), insulin (ELISA-Linco). Glucose levels were measured using a glucometer (Ascensia Elite).

### RNA extraction

The hippocampal tissue was processed using a Bead Beater (Bio-Spec, Bartlesville, OK) followed by RNA purification using the RNEasy Mini Kit (Qiagen, Valencia, CA) according to the manufacturer's instructions. The RNA was examined for quantity and quality using an Agilent Bioanalyzer 2100 (Agilent Technologies, Palo Alto, CA).

### Radioactive cDNA probe preparation and microarray hybridization

cDNA Probe preparation and microarray hybridization were performed as described previously [41]. Briefly, 5 µg total RNA was reverse-transcribed in a reaction mixture containing 8 µl of 5× first strand RT buffer, 1 µl of 1 µg/µl 12–18 mer poly (dT) primer, 4 µl of 20 mM dNTPs (-dCTP), 4 µl of 0.1 M DTT, 1 µl (40 U) of RNaseOUT, 6 µl of 3000 Ci/mmol α-^33^P-dCTP and DEPC-water to a final volume of 40 µl. The RT mixture was first heated at 65°C for 10 min, followed by incubation on ice for 2 min. Two microliters of Superscript II reverse transcriptase (Life Technologies, CA) was then added followed by incubation at 42°C for 35 min. One additional microliter of reverse transcriptase was added, followed by another 35 minute incubation. At the end of incubation, 5 µl of 0.5 M EDTA was added to chelate divalent cations. After addition of 10 µl of 1.0 M NaOH, the samples were incubated at 65°C for 30 min to hydrolyze the remaining RNA. Following the addition of 25 µl of 1 M Tris (pH 8.0), the samples were purified using Bio-Rad 6 purification columns (Hercules, CA). cDNA microarrays were pre-hybridized in a 4 ml hybridization buffer containing 3.2 ml Microhyb (Research Genetics, AL) and 0.8 ml 50% dextran sulfate, 10 µl of 10 mg/ml denatured human Cot 1 DNA (Life Technologies) and 10 µl of 8 mg/ml denatured poly(dA) (Pharmacia, NJ). After at least 4 h of pre-hybridization at 55°C, approximately 10^6^ cpm/ml of heat-denatured cDNA probes were added, followed by 17 h of incubation at 55°C. Hybridized arrays were washed in 2× SSC and 0.1% SDS once at room temperature followed by two washes in 2× SSC and 0.1% SDS at 65°C for 15 min each.

### Scanning and quantification

The microarrays were exposed to phosphorimager screens for 3 days. The screens were then scanned in a Molecular Dynamics STORM PhosphorImager (Sunnyvale, CA) at 50 µm resolution. Quantification of scanned screens was performed with ArrayPro software.

### Z-scores and z-ratio

Raw hybridization intensity data were log-transformed and normalized to yield z-scores, which in turn were used to calculate a z-ratio value for each gene with respect to the control tissues. The z-ratio was calculated as the difference between the observed gene z-scores for the experimental and the control comparisons, and dividing by the standard deviation associated with the distribution of these differences [42]. Z-ratio values ≥+2.0 or ≤−2.0 were chosen as cut-off values, defining increased and decreased expression, respectively.

### Filtering and cluster analysis

DIANE (NIH) software was used to filter the 17,000 genes. We filtered out genes which did not vary at least 1.25-fold from the log of the mean of the first filter in at least 60% of the genes expressed (p<0.01). Genes were clustered and sub-clusters were generated using DIANE software.

### Venn diagram generation

Multiple Venn diagrams were constructed that identified the genes that were either significantly up-regulated or significantly down-regulated compared to the *ad libitum* (control) rats. In addition to being significant at p<0.01, the changes needed to vary by greater than 25% from the controls using the median of the log value of the first filter.

### Gene pathway analyses

A complete set of 522 cellular pathways was obtained from the Molecular Signatures Database (MSigDB) created by the Broad Institute at the Massachusetts Institute of Technology [43]. The complete set was tested for Geneset enrichment using Parametric analysis of Gene set enrichment (PAGE, [18]). For each pathway a z-score was computed as previously described [44]. For each pathway z-score, a p-value was computed using JMP 6.0 software to test for the significance of the z-score obtained. These tools were part of DIANE 1.0 (see http://www.grc.nia.nih.gov/branches/rrb/dna/diane_software.pdf for information).

## Supporting Information

Table S1 Gene symbol and gene names.
(0.49 MB DOC)

Table S2 Description of the gene pathways significantly altered in figures 13 and 14.
(0.10 MB DOC)

## References

[1] McCay CM, Crowell MF, Maynard LA (1935). The effect of retarded growth upon the length of life-span and upon the ultimate body size.. J Nutr.

[2] Weindruch R, Walford RL (1988). Thomas, Charles, C. (Ed.) The Retardation of Aging and Disease by Dietary Restriction. Springfield, IL..

[3] Sprott RL (1997). Diet and calorie restriction.. Exp Gerontol.

[4] Chapman T, Partridge L (1996). Female fitness in *Drosophila melanogaster* and interaction between the effect of nutrition and of encounter rate with males.. Proc R Soc Lond Ser B Biol Sci.

[5] Houthoofd K, Braeckman BP, Lenaerts I, Brys K, De Vreese A (2002). Axonic growth up-regulates mass-specific metabolic rate, stress resistance, and extends life-span in *Caenorhabditis elegans*.. Exp Gerontol.

[6] Haslam DW, James WP (2005). Obesity.. Lancet.

[7] Levine AS, Billington CJ (1997). Why do we eat? A neural systems approach.. Annu Rev Nutr.

[8] Badman MK, Flier JS (2005). The gut and energy balance: visceral allies in the obesity wars.. Science.

[9] Volkow ND, Wise RA (2005). How can drug addiction help us understand obesity?. Nat Neurosci.

[10] Martin B, Pearson M, Kebejian L, Golden E, Keselman A (2007). Sex-dependent metabolic, neuroendocrine, and cognitive responses to dietary energy restriction and excess.. Endocrinology.

[11] Mattson MP, Duan W, Chan SL, Cheng A, Haughey N (2002). Neuroprotective and neurorestorative signal transduction mechanisms in brain aging: modification by genes, diet and behavior.. Neurobiol Aging.

[12] Diano S, Farr SA, Benoit SC, McNay EC, da Silva I (2006). Ghrelin controls hippocampal spine synapse density and memory performance.. Nat Neurosci.

[13] Elias M, Elias P, Sullivan L, Wolf P, D'Agostino R (2003). Lower cognitive function in the presence of obesity and hypertension: the Framingham heart study.. Int J Obes Relat Metab Disord.

[14] Greenwood C, Winocur G (2005). High-fat diets, insulin resistance and declining cognitive function.. Neurobiol Aging.

[15] Strupp B, Weingartner H, Kaye W, Gwirtsman H (1986). Cognitive processing in anorexia nervosa. A disturbance in automatic information processing.. Neuropsychobiology.

[16] Connan F, Murphy F, Connor S, Rich P, Murphy T (2006). Hippocampal volume and cognitive function in anorexia nervosa.. Psychiatry Res.

[17] Winocur G, Greenwood C, Piroli G, Grillo C, Reznikov L (2005). Memory impairment in obese Zucker rats: an investigation of cognitive function in an animal model of insulin resistance and obesity.. Behav Neurosci.

[18] Kim SY, Volsky DJ (2005). PAGE: parametric analysis of gene set enrichment.. BMC Bioinformatics..

[19] Pappenberger G, McCormack EA, Willison KR (2006). Quantitative actin folding reactions using yeast CCT purified via an internal tag in the CCT3/gamma subunit.. J Mol Biol.

[20] Gatward N (2000). Anorexia nervosa: an evolutionary puzzle.. Eur Eat Disord Rev.

[21] Magurie ER, Gadian DG, Johnsrude IS, Good CD, Ashburner J (2000). Navigation-related structural change in the hippocampus of taxi drivers.. Proc Natl Acad Sci U S A.

[22] Pereira AC, Huddleston DE, Brickman AM, Sosunov AA, Hen R (2007). An in vivo correlate of exercise-induced neurogenesis in the adult dentate gyrus.. Proc Natl Acad Sci U S A.

[23] Liu B, Liao J, Rao X, Kushner SA, Chung CD (1998). Inhibition of STAT1-mediated gene activation by PIAS1.. Proc Natl Acad Sci U S A.

[24] Muller S, Hoege C, Pyrowolakis G, Jentsch S (2001). SUMO, ubiquitin's mysterious cousin.. Nat Rev Mol Cell Biol.

[25] Tang Z, El Far O, Betz H, Scheschonka A (2005). Pias1 interaction and sumoylation of metabotropic glutamate receptor 8.. J Biol Chem.

[26] Melchior F, Schegraut M, Pichler A (2003). SUMO: ligases, isopeptidases and nuclear pores.. Trends Biochem Sci.

[27] Song J, Durrin LK, Wilkinson TA, Krontiris TG, Chen Y (2004). Identification of a sumo-binding motif that recognizes sumo-modified proteins.. Proc Natl Acad Sci U S A.

[28] Mahmoud GS, Grover LM (2006). Growth hormone enhances excitatory synaptic transmission in area CA1 of rat hippocampus.. J Neurophysiol.

[29] Ross JL (2005). Effects of growth hormone on cognitive function.. Horm Res.

[30] Phillips GR, Anderson TR, Florens L, Gudas C, Magda G (2004). Actin-binding proteins in a post-synaptic preparation: Lasp-1 is a component of central nervous system synapses and dendritic spines.. J Neurosci Res.

[31] Ferguson GD, Wang H, Herschmann HR, Storm DR (2004). Altered hippocampal short-term plasticity and associative memory in synaptotagmin IV (-/-) mice.. Hippocampus.

[32] Antunes-Martins A, Mizuno K, Irvine EE, Lepicard EM, Glese KP (2007). Sex-dependent up-regulation of two splicing factors, Psf and SrRp20, during hippocampal memory formation.. Learn Mem.

[33] Lee CK, Weindruch R, Prolla TA (2000). Gene-expression profile of the ageing brain in mice.. Nat Genet.

[34] Mattson MP (2003). Excitotoxic and excitoprotective mechanisms: abundant targets for the prevention and treatment of neurodegenerative disorders.. Neuromolecular Med.

[35] Lu T, Pan Y, Kao SY, Li C, Kohane I (2004). Gene regulation and DNA damage in the ageing human brain.. Nature.

[36] Blalock EM, Geddes JW, Chen KC, Porter NM, Markesbery WR (2004). Incipient Alzheimer's disease: microarray correlation analyses reveal major transcriptional and tumor suppressor responses.. Proc Natl Acad Sci U S A.

[37] Cavallaro S, D'Agata V, Manickam P, Dufour F, Alkon DL (2002). Memory-specific temporal profiles of gene expression in the hippocampus.. Proc Natl Acad Sci U S A.

[38] Perreau VM, Adlard PA, Anderson AJ, Cotman CW (2005). Exercise-induced gene expression changes in the rat spinal cord.. Gene Expr.

[39] Bahar R, Hartmann CH, Rodriguez KA, Denny AD, Busuttil RA (2006). Increased cell-to-cell variation in gene expression in ageing mouse heart.. Nature.

[40] Xu X, Zhan M, Duan W, Prabhu V, Brenneman R (2007). Gene expression atlas of the mouse central nervous system: impact and interactions of age, energy intake and gender.. Genome Biol.

[41] Whitney LW, Becker KG, Tresser NJ, Caballero-Ramos CI, Munson PJ (1999). Analysis of gene expression in multiple sclerosis lesions using cDNA microarrays.. Ann Neurol.

[42] Cheadle C, Cho-Chung YS, Becker KG, Vawter MP (2003). Application of z-score transformation to Affymetrix data.. Appl Bioinformatics.

[43] Subramanian A, Tamayo P, Mootha VK, Mukherjee S, Ebert BL (2005). Gene set enrichment analysis: a knowledge-based approach for interpreting genome-wide expression profiles.. Proc Natl Acad Sci U S A.

[44] Baur JA, Pearson KJ, Price NL, Jamieson HA, Lerin C (2006). Resveratrol improves health and survival of mice on a high-calorie diet.. Nature.

