# Supplementary material for: Conserved and Differential Effects of Dietary Energy Intake on the Hippocampal Transcriptomes of Females and Males
Source: PLoS One. 2008 Jun 11;3(6):e2398. doi: 10.1371/journal.pone.0002398 (PMC2405949; doi:10.1371/journal.pone.0002398)
Supplement: Table S1 — Gene symbol and gene names. (0.49 MB DOC) [file pone.0002398.s001.doc]

**Supplementary Table 1: Gene symbol and gene names.**

| **Gene Symbol** | **Gene Name** |
| --- | --- |
| 10-Sep | septin 10 |
| 1810063B07Rik | DNA segment, Chr 14, ERATO Doi 170, expressed |
| 2210408E11Rik | myocyte enhancer factor 2D |
| 2310045H08Rik | methyltransferase Cyt19 |
| 2410016F01Rik | hypothetical protein, clone 2-24 |
| 2700050L05Rik | Mus musculus, Similar to hypothetical protein FLJ21617 |
| 2900010J23Rik | expressed sequence AI046671 |
| 4632411B12Rik | RIKEN cDNA 4632411B12 gene |
| 4732418C07Rik | RIKEN cDNA 4732418C07 gene |
| 4921515A04Rik | expressed sequence AA408298 |
| 5730472N09Rik | expressed sequence AA408683 |
| 6430706D22Rik | Mus musculus, clone MGC:7054 IMAGE:3156506 |
| 6620401K05Rik | RIKEN cDNA 6620401K05 gene |
| 9030624J02Rik | RIKEN cDNA 9030624J02 gene |
| 9530010C24Rik | RIKEN cDNA 9530010C24 gene |
| 9630050M13Rik | Mus musculus, clone IMAGE:5375863 |
| A230054D04Rik | RIKEN cDNA A230054D04 gene |
| A430005L14Rik | expressed sequence C79672 |
| A530058O07Rik | RIKEN cDNA A530058O07 gene |
| A730082K24Rik | RIKEN cDNA A730082K24 gene |
| A930034L06Rik | expressed sequence AL022637 |
| AA407107 | expressed sequence AA407107 |
| AA408213 | expressed sequence AA408213 |
| AA536743 | Mus musculus, Similar to hypothetical gene LOC133157 |
| AA589532 | expressed sequence AA589532 |
| Abcb1b | ATP-binding cassette, sub-family B (MDR/TAP), member 1B |
| Abcc3 | ATP-binding cassette, sub-family C (CFTR/MRP), member 3 |
| Abi1 | spectrin SH3 domain binding protein 1 |
| Ablim1 | actin-binding LIM protein 1 |
| Abr | active BCR-related gene |
| Abtb2 | ankyrin repeat and BTB (POZ) domain containing 2 |
| Acsl4 | acyl-CoA synthetase long-chain family member 4 |
| Actb | actin, beta, cytoplasmic |
| Actr10 | actin-related protein 11 homolog |
| Adam17 | a disintegrin and metalloproteinase domain 17 |
| Adamts10 | a disintegrin-like and metallopeptidase (reprolysin type) with thrombospondin type 1 motif, 10 |
| Adamts19 | a disintegrin-like and metallopeptidase (reprolysin type) with thrombospondin type 1 motif, 19 |
| Adnp | activity-dependent neuroprotective protein |
| Adsl | adenylosuccinate lyase |
| Agpat1 | 1-acylglycerol-3-phosphate O-acyltransferase 1 (lysophosphatidic acid acyltransferase, alpha) |
| AI303526 | expressed sequence AI303526 |
| AI462493 | expressed sequence AI462493 |
| AI467481 | expressed sequence AI467481 |
| AI481105 | Mus musculus, Similar to KIAA1404 protein |
| AI987944 | expressed sequence AI987944 |
| Akap9 | A kinase (PRKA) anchor protein (yotiao) 9 |
| Akr1c13 | aldo-keto reductase family 1, member C13 |
| Alad | aminolevulinate, delta-, dehydratase |
| Alb1 | albumin 1 |
| Aldh1b1 | aldehyde dehydrogenase 1 family, member B1 |
| Aldoa | aldolase 1, A isoform |
| Ampd3 | AMP deaminase 3 |
| Anp32b | acidic nuclear phosphoprotein 32 family, member B |
| Ap2a1 | adaptor protein complex AP-2, alpha 1 subunit |
| Ap3s2 | peroxisomal biogenesis factor 11a |
| Apg5l | autophagy 5-like |
| Aph1a | carbonic anhydrase 14 |
| Apoa1 | apolipoprotein A-I |
| Aprt | adenine phosphoribosyl transferase |
| Armet | arginine-rich, mutated in early stage tumors |
| Arpc2 | Bcl2-interacting killer-like |
| Arpp19 | cyclic AMP phosphoprotein, 19 kDa |
| As3mt | methyltransferase Cyt19 |
| Atp5a1 | ATP synthase, H+ transporting, mitochondrial F1 complex, alpha subunit, isoform 1 |
| Atp5g1 | ATP synthase, H+ transporting, mitochondrial F0 complex, subunit c (subunit 9), isoform 1 |
| Atp5j2 | ATP synthase, H+ transporting, mitochondrial F0 complex, subunit f, isoform 2 |
| Atpaf2 | ATP synthase mitochondrial F1 complex assembly factor 2 |
| Atrx | alpha thalassemia/mental retardation syndrome X-linked homolog |
| AU014965 | expressed sequence AU014965 |
| AU015584 | expressed sequence AU015584 |
| AU015738 | expressed sequence AU015738 |
| AU015892 | expressed sequence AU015892 |
| AU018728 | expressed sequence AU018728 |
| AU019278 | expressed sequence AU019278 |
| AU020745 | expressed sequence AU020745 |
| AU021725 | expressed sequence AU021725 |
| AU022252 | expressed sequence AU022252 |
| AU022554 | expressed sequence AU022554 |
| AW549542 | expressed sequence AW549542 |
| AW549877 | Mus musculus, clone MGC:12113 |
| B230379M23Rik | Mus musculus, clone IMAGE:3586067 |
| B3galt4 | UDP-Gal:betaGlcNAc beta 1,3-galactosyltransferase, polypeptide 4 |
| B4galt7 | Mus musculus, Similar to xylosylprotein beta1,4-galactosyltransferaseansferase I) |
| Banp | Btg3 associated nuclear protein |
| BC002230 | Mus musculus, Similar to hypothetical protein FLJ10008 |
| BC003940 | phosphotyrosyl phosphatase activator |
| BC011248 | Mus musculus, clone MGC:19067 IMAGE:4192711 |
| Bcat1 | branched chain aminotransferase 1, cytosolic |
| Becn1 | beclin 1 (coiled-coil, myosin-like BCL2-interacting protein) |
| Birc2 | baculoviral IAP repeat-containing 2 |
| Blcap | bladder cancer associated protein homolog |
| Bmp1 | bone morphogenetic protein 1 |
| Bpnt1 | bisphosphate 3'-nucleotidase 1 |
| Braf | Braf transforming gene |
| Brd2 | bromodomain-containing 2 |
| C030046I01Rik | RIKEN cDNA C030046I01 gene |
| C130032J12Rik | expressed sequence AW047581 |
| C130061O14Rik | RIKEN cDNA C130061O14 gene |
| C76294 | expressed sequence C76294 |
| C76332 | expressed sequence C76332 |
| C76554 | expressed sequence C76554 |
| C77591 | expressed sequence C77591 |
| C78297 | expressed sequence C78297 |
| C78409 | expressed sequence C78409 |
| C78444 | expressed sequence C78444 |
| C78505 | expressed sequence C78505 |
| C78532 | expressed sequence C78532 |
| C78859 | expressed sequence C78859 |
| C78880 | expressed sequence C78880 |
| C79238 | expressed sequence C79238 |
| C79491 | expressed sequence C79491 |
| C79601 | expressed sequence C79601 |
| C79657 | expressed sequence C79657 |
| C79946 | expressed sequence C79946 |
| C80113 | expressed sequence C80113 |
| C80283 | expressed sequence C80283 |
| C80406 | expressed sequence C80406 |
| C80425 | expressed sequence C80425 |
| C80446 | expressed sequence C80446 |
| C80571 | expressed sequence C80571 |
| C80914 | expressed sequence C80914 |
| C80918 | expressed sequence C80918 |
| C80993 | expressed sequence C80993 |
| C81364 | expressed sequence C81364 |
| C81543 | expressed sequence C81543 |
| C81608 | expressed sequence C81608 |
| C86400 | expressed sequence C86400 |
| C86544 | expressed sequence C86544 |
| C86753 | expressed sequence C86753 |
| C86896 | expressed sequence C86896 |
| C87011 | expressed sequence C87011 |
| C87482 | expressed sequence C87482 |
| Cacnb3 | calcium channel, voltage-dependent, beta 3 subunit |
| Calm2 | calmodulin 2 |
| Caml | calcium modulating ligand |
| Cap1 | adenylyl cyclase-associated CAP protein homolog 1 |
| Catns | catenin src |
| Cbx5 | chromobox homolog 5 |
| Ccdc117 | coiled-coil domain containing 117 |
| Ccnd2 | cyclin D2 |
| Ccng1 | cyclin G |
| Ccnl1 | cyclin L1 |
| Cct3 | chaperonin subunit 3 (gamma) |
| Cct8 | chaperonin subunit 8 (theta) |
| Cd84 | CD84 antigen |
| Cd9 | CD9 antigen |
| Cdc25c | cell division cycle 25 homolog C |
| Cdc34 | cell division cycle 34 homolog |
| Cdc6 | cell division cycle 6 homolog |
| Cdca3 | gene rich cluster, C8 gene |
| Cdipt | CDP-diacylglycerol--inositol 3-phosphatidyltransferase (phosphatidylinositol synthase) |
| Ceacam9 | CEA-related cell adhesion molecule 9 |
| Cebpa-rs1 | CCAAT/enhancer binding protein alpha (C/EBP), related sequence 1 |
| Cebpz | CCAAT/enhancer binding protein alpha (C/EBP), related sequence 1 |
| Cep110 | centrosomal protein 110 |
| Chaf1a | chromatin assembly factor 1, subunit A (p150) |
| Chchd2 | coiled-coil-helix-coiled-coil-helix domain containing 2 |
| Chordc1 | cysteine and histidine-rich domain (CHORD)-containing, zinc-binding protein 1 |
| Chtf18 | CTF18, chromosome transmission fidelity factor 18 homolog |
| Ckmt1 | creatine kinase, mitochondrial 1, ubiquitous |
| Clk | CDC-like kinase |
| Clstn1 | calsyntenin 1 |
| Cnot2 | CCR4-NOT transcription complex, subunit 2 |
| Col2a1 | procollagen, type II, alpha 1 |
| Col4a1 | procollagen, type IV, alpha 1 |
| Col5a2 | procollagen, type V, alpha 2 |
| Copb2 | coatomer protein complex, subunit beta 2 (beta prime) |
| Cops3 | COP9 (constitutive photomorphogenic) homolog, subunit 3 (Arabidopsis thaliana) |
| Coro1b | coronin, actin binding protein 1B |
| Cox7a2l | cytochrome c oxidase subunit VIIa polypeptide 2-like |
| Cpeb1 | cytoplasmic polyadenylation element binding protein |
| Cpne3 | copine III |
| Cpox | coproporphyrinogen oxidase |
| Crk | avian sarcoma virus CT10 (v-crk) oncogene homolog |
| Crkrs | Cdc2-related kinase, arginine/serine-rich |
| Cryab | crystallin, alpha B |
| Csda | Y box protein 3 |
| Csde1 | Mus musculus, clone MGC:19174 IMAGE:4224466 |
| Csh1 | chorionic somatomammotropin hormone 1 |
| Csnk1e | casein kinase 1, epsilon |
| Csnk2b | casein kinase II, beta subunit |
| Cts8 | cathepsin 8 |
| Ctsd | cathepsin D |
| Ctsz | cathepsin Z |
| Cugbp1 | CUG triplet repeat, RNA binding protein 1 |
| Cul4b | cullin 4B |
| Cybb | cytochrome b-245, beta polypeptide |
| Cyp17a1 | cytochrome P450, 17 |
| D030041N15Rik | trans-acting transcription factor 1 |
| D030054H19Rik | RIKEN cDNA D030054H19 gene |
| D10Ertd447e | DNA segment, Chr 10, ERATO Doi 447, expressed |
| D10Ertd709e | DNA segment, Chr 10, ERATO Doi 709, expressed |
| D10Wsu102e | DNA segment, Chr 10, Wayne State University 102, expressed |
| D15Ertd180e | DNA segment, Chr 15, ERATO Doi 180, expressed |
| D15Ertd320e | DNA segment, Chr 15, ERATO Doi 320, expressed |
| D15Ertd492e | DNA segment, Chr 15, ERATO Doi 492, expressed |
| D16Ertd778e | DNA segment, Chr 16, ERATO Doi 778, expressed |
| D16Ertd88e | DNA segment, Chr 16, ERATO Doi 88, expressed |
| D16Wsu65e | DNA segment, Chr 16, Wayne State University 65, expressed |
| D17Ertd657e | DNA segment, Chr 17, ERATO Doi 657, expressed |
| D19Ertd200e | DNA segment, Chr 19, ERATO Doi 200, expressed |
| D19Wsu162e | DNA segment, Chr 19, Wayne State University 162, expressed |
| D1Ertd185e | DNA segment, Chr 1, ERATO Doi 185, expressed |
| D1Ertd309e | DNA segment, Chr 1, ERATO Doi 309, expressed |
| D2Ertd329e | DNA segment, Chr 2, ERATO Doi 329, expressed |
| D2Ertd337e | DNA segment, Chr 2, ERATO Doi 337, expressed |
| D2Ertd92e | DNA segment, Chr 2, ERATO Doi 92, expressed |
| D3Ucla1 | DNA segment, Chr 3, University of California at Los Angeles 1 |
| D4Ertd103e | DNA segment, Chr 4, ERATO Doi 103, expressed |
| D4Ertd111e | DNA segment, Chr 4, ERATO Doi 111, expressed |
| D4Ertd429e | DNA segment, Chr 4, ERATO Doi 429, expressed |
| D4Ertd58e | DNA segment, Chr 4, ERATO Doi 58, expressed |
| D5Ertd255e | DNA segment, Chr 5, ERATO Doi 255, expressed |
| D5Ertd77e | DNA segment, Chr 5, ERATO Doi 77, expressed |
| D5Wsu178e | DNA segment, Chr 5, Wayne State University 178, expressed |
| D6Ertd469e | DNA segment, Chr 6, ERATO Doi 469, expressed |
| D6Ertd87e | DNA segment, Chr 6, ERATO Doi 87, expressed |
| D7Ertd193e | DNA segment, Chr 7, ERATO Doi 193, expressed |
| D7Ertd413e | DNA segment, Chr 7, ERATO Doi 413, expressed |
| D7Ertd443e | DNA segment, Chr 7, ERATO Doi 443, expressed |
| D7Ertd715e | DNA segment, Chr 7, ERATO Doi 715, expressed |
| D8Ertd107e | DNA segment, Chr 8, ERATO Doi 107, expressed |
| D8Ertd294e | DNA segment, Chr 8, ERATO Doi 294, expressed |
| D8Ertd457e | DNA segment, Chr 8, ERATO Doi 457, expressed |
| D8Ertd575e | DNA segment, Chr 8, ERATO Doi 575, expressed |
| Dazap1 | DAZ associated protein 1 |
| Dazl | deleted in azoospermia-like |
| Dbf4 | DBF4 homolog |
| Dbnl | drebrin-like |
| Dcbld2 | discoidin, CUB and LCCL domain containing 2 |
| Dci | dodecenoyl-Coenzyme A delta isomerase (3,2 trans-enoyl-Coenyme A isomerase) |
| Ddx19b | DEAD/H (Asp-Glu-Ala-Asp/His) box polypeptide 19 |
| Dgke | diacylglycerol kinase, epsilon |
| Dhcr7 | 7-dehydrocholesterol reductase |
| Dhfr | dihydrofolate reductase |
| Dhx15 | DEAD/H (Asp-Glu-Ala-Asp/His) box polypeptide 15 (RNA helicase A) |
| Dhx36 | DEAH (Asp-Glu-Ala-His) box polypeptide 36 |
| Dnajb6 | DnaJ (Hsp40) homolog, subfamily B, member 6 |
| Dnchc1 | dynein, cytoplasmic, heavy chain 1 |
| Dstn | destrin |
| DXErtd223e | DNA segment, Chr X, ERATO Doi 223, expressed |
| Dynll2 | dynein light chain LC8-type 2 |
| Dysf | Mus musculus, Similar to dysferlin, clone IMAGE:5324940 |
| E2f8 | E2F transcription factor 8 |
| Eef2 | eukaryotic translation elongation factor 2 |
| Ehd1 | EH-domain containing 1 |
| Eif1a | eukaryotic translation initiation factor 1A |
| Eif2s1 | eukaryotic translation initiation factor 2A |
| Eif2s2 | eukaryotic translation initiation factor 2, subunit 2 (beta, 38kDa) |
| Eif3s10 | eukaryotic translation initiation factor 3 |
| Eif3s2 | eukaryotic translation initiation factor 3, subunit 2 (beta, 36kD) |
| Eif3s6 | mammary tumor integration site 6 |
| Entpd2 | ectonucleoside triphosphate diphosphohydrolase 2 |
| Ephx2 | epoxide hydrolase 2, cytoplasmic |
| Epn2 | epsin 2 |
| Eppb9 | endothelial precursor protein B9 |
| Eprs | M.musculus mRNA for glutamyl-tRNA synthetase |
| Ern1 | endoplasmic reticulum (ER) to nucleus signalling 1 |
| Errfi1 | ERBB receptor feedback inhibitor 1 |
| Espn | espin |
| Falz | Mus musculus, clone IMAGE:3487914 |
| Fastk | Fas-activated serine/threonine kinase |
| Fbxo6 | f-box only protein 6b |
| Fdft1 | farnesyl diphosphate farnesyl transferase 1 |
| Fgf10 | fibroblast growth factor 10 |
| Fignl1 | fidgetin-like 1 |
| Fkbp4 | FK506 binding protein 4 (59 kDa) |
| Fliih | flightless I homolog (Drosophila) |
| Fndc3b | fibronectin type III domain containing 3B |
| Fndc6 | fibronectin type III domain containing 6 |
| Foxj2 | forkhead box J2 |
| Frag1 | FGF receptor activating protein 1 |
| Frk | B-cell src-homology tyrosine kinase |
| Fzd7 | frizzled homolog 7 |
| G3bp | Ras-GTPase-activating protein SH3-domain binding protein |
| Gadd45g | growth arrest and DNA-damage-inducible 45 gamma |
| Gcnt2 | glucosaminyl (N-acetyl) transferase 2, I-branching enzyme |
| Gdf9 | growth differentiation factor 9 |
| Gjb5 | gap junction membrane channel protein beta 5 |
| Glg1 | selectin, endothelial cell, ligand |
| Gna13 | guanine nucleotide binding protein, alpha 13 |
| Gnpat | glyceronephosphate O-acyltransferase |
| Gpc3 | glypican 3 |
| Gpi1 | glucose phosphate isomerase 1 complex |
| Gpr37 | G protein-coupled receptor 37 |
| Grtp1 | GH regulated TBC protein 1 |
| Gss | glutathione synthetase |
| Gtf2i | general transcription factor II I |
| Gtf3c1 | general transcription factor III C 1 |
| Gtl6 | expressed sequence C78997 |
| Gtse1 | G two S phase expressed protein 1 |
| H19 | H19 fetal liver mRNA |
| H2afz | H2A histone family, member Z |
| Hax1 | HS1 binding protein |
| Hba-a1 | hemoglobin alpha, adult chain 1 |
| Hbb-b1 | hemoglobin alpha, adult chain 1 |
| Hbb-y | hemoglobin Y, beta-like embryonic chain |
| Hdgf | hepatoma-derived growth factor |
| Heatr1 | HEAT repeat containing 1 |
| Hexb | hexosaminidase B |
| Hint1 | histidine triad nucleotide binding protein |
| Hist1h2ao | Mus musculus, similar to H2A histone family |
| Hmox1 | heme oxygenase (decycling) 1 |
| Hn1 | hematological and neurological expressed sequence 1 |
| Hnrpa2b1 | heterogeneous nuclear ribonucleoprotein A2/B1 |
| Hnrpul1 | heterogeneous nuclear ribonucleoprotein U-like 1 |
| Hsd17b4 | hydroxysteroid 17-beta dehydrogenase 4 |
| Hsp90ab1 | heat shock protein, 84 kDa 1 |
| Hspa8 | heat shock 70kD protein 8 |
| Hunk | hormonally upregulated Neu-associated kinase |
| Iars | isoleucine-tRNA synthetase |
| Icmt | isoprenylcysteine carboxyl methyltransferase |
| Idh2 | isocitrate dehydrogenase 2 (NADP+), mitochondrial |
| Ifrg15 | interferon alpha responsive gene, 15 kDa |
| Igk-V8 | DNA segment, Chr 6, ERATO Doi 456, expressed |
| Il1rl2 | expressed sequence AI481289 |
| Immt | inner membrane protein, mitochondrial |
| Ing1 | inhibitor of growth family, member 1 |
| Inpp5e | inositol polyphosphate-5-phosphatase, 72 kDa |
| Irf1 | interferon regulatory factor 1 |
| Itfg2 | integrin alpha FG-GAP repeat containing 2 |
| Itm2c | integral membrane protein 3 |
| Itpr5 | inositol 1,4,5-triphosphate receptor 5 |
| Jak2 | Janus kinase 2 |
| Jrk | jerky |
| Kars | lysyl-tRNA synthetase |
| Kbtbd8 | kelch repeat and BTB (POZ) domain containing 8 |
| Kcnk6 | potassium inwardly-rectifying channel, subfamily K, member 6 |
| Kcnn2 | potassium intermediate/small conductance calcium-activated channel |
| Kctd5 | lipoprotein lipase |
| Kif5b | kinesin family member 5B |
| Klf6 | Kruppel-like factor 6 |
| Klhl2 | kelch-like 2, Mayven (Drosophila) |
| Kpna6 | Mus musculus, clone IMAGE:4236601 |
| Kpnb3 | karyopherin (importin) beta 3 |
| Kremen1 | kringle containing transmembrane protein 1 |
| Krt7 | keratin 7 |
| Krt8 | keratin complex 2, basic, gene 8 |
| L3mbtl3 | expressed sequence AI481284 |
| Larp2 | La ribonucleoprotein domain family, member 2 |
| Lars | leucyl-tRNA synthetase |
| Lasp1 | LIM and SH3 protein 1 |
| Lcp1 | plastin 2, L |
| Lgals1 | lectin, galactose binding, soluble 1 |
| Lgals3 | lectin, galactose binding, soluble 3 |
| Lgals8 | lectin, galactose binding, soluble 8 |
| Lima1 | LIM domain and actin binding 1 |
| Limd1 | LIM domains containing 1 |
| Lims2 | Mus musculus, Similar to hypothetical protein FLJ10044 |
| Lmnb1 | lamin B1 |
| LOC215736 | similar to sodium/bile acid co-transporter |
| Lt1 | lurcher transcript 1 |
| Ltf | lactotransferrin |
| Luc7l2 | LUC7-like 2 |
| Man1b | mannosidase 1, beta |
| Man2b1 | mannosidase 2, alpha B1 |
| Mapk13 | mitogen activated protein kinase 13 |
| March_7 | axotrophin |
| Mark2 | ELKL motif kinase |
| Mbd3 | methyl-CpG binding domain protein 3 |
| Mbd6 | methyl-CpG binding domain protein 6 |
| Mcm3 | mini chromosome maintenance deficient |
| Mcm3ap | minichromosome maintenance deficient 3-associated protein |
| Mcrs1 | microspherule protein 1 |
| Mdh1 | malate dehydrogenase, soluble |
| Mef2d | myocyte enhancer factor 2D |
| Mettl2 | DNA segment, Chr 11, ERATO Doi 768, expressed |
| Mfap3 | microfibrillar-associated protein 3 |
| Mgat4b | mannoside acetylglucosaminyltransferase 4, isoenzyme B |
| MGC18837 | RAB3D, member RAS oncogene family |
| Mocs1 | molybdenum cofactor synthesis 1 |
| Morf4l1 | testis expressed gene 189 |
| Mpv17 | Mpv17 transgene, kidney disease mutant |
| Mrpl4 | mitochondrial ribosomal protein L4 |
| Mrps15 | mitochondrial ribosomal protein S15 |
| Mrps25 | mitochondrial ribosomal protein S25 |
| Mthfd1 | methylenetetrahydrofolate dehydrogenase (NADP+ dependent) |
| Mtpn | granule cell differentiation protein |
| Nalp6 | DNA segment, Chr 7, ERATO Doi 187, expressed |
| Nans | Mus musculus sialic acid synthase (Sas) |
| Napa | N-ethylmaleimide sensitive fusion protein attachment protein alpha |
| Narg1 | NMDA receptor-regulated gene 1 |
| Ncl | nucleolin |
| Ncoa6 | nuclear receptor coactivator 6 |
| Ncstn | nicastrin |
| Ndfip1 | Nedd4 WW binding protein 5 |
| Ndn | necdin |
| Ndst2 | N-deacetylase/N-sulfotransferase (heparan glucosaminyl) 2 |
| Nek7 | NIMA (never in mitosis gene a)-related expressed kinase 7 |
| Net1 | neuroepithelial cell transforming gene 1 |
| Neu1 | neuraminidase 1 |
| Nfe2l2 | nuclear, factor, erythroid derived 2, like 2 |
| Nid1 | nidogen 1 |
| Nmt1 | N-myristoyltransferase 1 |
| Nolc1 | nucleolar and coiled-body phosphoprotein 1 |
| Nras | neuroblastoma ras oncogene |
| Nsbp1 | nucleosome binding protein 1 |
| Nsf | N-ethylmaleimide sensitive fusion protein |
| Nucb2 | nucleobindin 2 |
| Nup160 | gene trap locus 1-13 |
| Nupr1 | nuclear protein 1 |
| Nxf1 | nuclear RNA export factor 1 homolog |
| Oaz1 | ornithine decarboxylase antizyme |
| Onecut1 | one cut domain, family member 1 |
| Ovgp1 | oviductal glycoprotein 1, 120kD |
| Parp12 | poly (ADP-ribose) polymerase family, member 12 |
| Paxip1 | PAX interacting (with transcription-activation domain) protein 1 |
| Pcbp2 | poly(rC) binding protein 2 |
| Pcdh7 | protocadherin 7 |
| Pcna | proliferating cell nuclear antigen |
| Pcsk5 | proprotein convertase subtilisin/kexin type 5 |
| Pdap1 | tyrosine 3-monooxygenase/tryptophan 5-monooxygenase activation protein |
| Pdcd5 | programmed cell death 5 |
| Pde8a | phosphodiesterase 8A |
| Pdzd3 | natrium-phosphate cotransporter IIa C-terminal-associated protein 2 |
| Pdzk2 | natrium-phosphate cotransporter IIa C-terminal-associated protein 2 |
| Peli1 | pellino 1 |
| Perp | p53 apoptosis effector related to Pmp22 |
| Pex11a | peroxisomal biogenesis factor 11a |
| Pggt1b | protein geranylgeranyltransferase type I, beta subunit |
| Pgrmc1 | progesterone receptor membrane component 1 |
| Phf17 | PHD finger protein 17 |
| Phgdh | 3-phosphoglycerate dehydrogenase |
| Phtf1 | putative homeodomain transcription factor |
| Pias1 | protein inhibitor of activated STAT 1 |
| Pik3cd | phosphatidylinositol 3-kinase catalytic delta polypeptide |
| Pinx1 | PIN2/TRF1-interacting protein |
| Pisd | Mus musculus, Similar to phosphatidylserine decarboxylase, mRNA, complete cds |
| Plekha4 | pleckstrin homology domain containing, family A (phosphoinositide binding specific) |
| Plekha5 | pleckstrin homology domain containing, family A member 5 |
| Plekhb2 | Mus musculus evectin-2 (Evt2) mRNA, complete cds |
| Plxnb1 | plexin B1 |
| Pnp | purine-nucleoside phosphorylase |
| Pnpo | pyridoxine 5'-phosphate oxidase |
| Pou5f1 | POU domain, class 5, transcription factor 1 |
| Ppp1r14b | phospholipase c neighboring |
| Ppp2ca | protein phosphatase 2a, catalytic subunit, alpha isoform |
| Ppp2r4 | phosphotyrosyl phosphatase activator |
| Ppp4c | protein phosphatase 4, catalytic subunit |
| Ppt1 | palmitoyl-protein thioesterase |
| Prdx6 | peroxiredoxin 5 |
| Prkra | protein kinase, interferon inducible double stranded RNA dependent activator |
| Prlpa | prolactin-like protein A |
| Prom1 | prominin 1 |
| Prpf4b | pre-mRNA protein kinase |
| Prps1 | phosphoribosyl pyrophosphate synthetase 1 |
| Prss25 | ancient ubiquitous protein |
| Psa | puromycin-sensitive aminopeptidase |
| Psap | prosaposin |
| Psma7 | proteasome (prosome, macropain) subunit, alpha type 7 |
| Psmc5 | protease (prosome, macropain) 26S subunit, ATPase 5 |
| Ptp4a2 | protein tyrosine phosphatase 4a2 |
| Pvrl2 | Mus musculus poliovirus sensitivity (Pvs), mRNA |
| Rab14 | RAB14, member RAS oncogene family |
| Rab8a | cell line NK14 derived transforming oncogene |
| Rabggtb | RAB geranylgeranyl transferase, b subunit |
| Raly | hnRNP-associated with lethal yellow |
| Ranbp1 | RAN binding protein 1 |
| Rbm26 | RNA binding motif protein 26 |
| Rbm27 | RNA binding motif protein 27 |
| Rbm28 | RNA binding motif protein 28 |
| Rbm3 | RNA binding motif protein 3 |
| Rbm9 | fox-1 homolog (C. elegans) |
| Rex3 | reduced expression 3 |
| Rfxank | regulatory factor X-associated ankyrin-containing protein |
| Rgs2 | regulator of G-protein signaling 2 |
| Rin3 | Ras and Rab interactor 3 |
| Riok3 | sudD, suppressor of bimD6 homolog |
| Rnf111 | arkadia |
| Rnf12 | ring finger protein 12 |
| Robo1 | roundabout homolog 1 |
| Rpl18 | ribosomal protein L18 |
| Rpl23 | ribosomal protein L23 |
| Rpl26 | ribosomal protein L26 |
| Rpl27 | ribosomal protein L27 |
| Rpl27a | ribosomal protein L27a |
| Rpl28 | ribosomal protein L28 |
| Rpl5 | ribosomal protein L5 |
| Rpl7 | ribosomal protein L7 |
| Rpl8 | ribosomal protein L8 |
| Rpo1-4 | RNA polymerase 1-4 (194 kDa subunit) |
| Rps14 | ribosomal protein S14 |
| Rps15 | ribosomal protein S15 |
| Rps18 | ribosomal protein S18 |
| Rps6ka1 | ribosomal protein S6 kinase polypeptide 1 |
| Rrbp1 | ribosome binding protein 1 |
| Rrm2 | ribonucleotide reductase M2 |
| Runx2 | runt related transcription factor 2 |
| S100a13 | S100 calcium binding protein A13 |
| Sae1 | ubiquitin-like 1 (sentrin) activating enzyme E1A |
| Sara1 | SAR1a gene homolog |
| Sbk1 | SH3-binding kinase 1 |
| Scd2 | stearoyl-Coenzyme A desaturase 2 |
| Scg3 | secretogranin III |
| Sec61a1 | SEC61, alpha subunit |
| Sema3e | sema domain, immunoglobulin domain (Ig), short basic domain, secreted, (semaphorin) 3E |
| Sema3f | sema domain, immunoglobulin domain (Ig), short basic domain, secreted, (semaphorin) 3 F |
| Serpinb9f | serine protease inhibitor 13 |
| Serpine2 | serine (or cysteine) proteinase inhibitor, clade E (nexin, plasminogen activator inhibitor type 1) |
| Set | SET translocation |
| Sf3a2 | splicing factor 3a, subunit 2, 66kD |
| Sfmbt2 | DNA segment, Chr 2, Wayne State University 23, expressed |
| Sfn | makorin, ring finger protein, 3 |
| Sfrs10 | silica-induced gene 41 |
| Sfrs3 | splicing factor, arginine/serine-rich 3 (SRp20) |
| Sfxn1 | sideroflexin 1 |
| Sh2bp1 | TPR-containing, SH2-binding phosphoprotein |
| Sh3gl2 | SH3 domain protein 2A |
| Sh3glb1 | SH3-domain GRB2-like B1 (endophilin) |
| Shmt1 | serine hydroxymethyl transferase 1 (soluble) |
| Shoc2 | soc-2 (suppressor of clear) homolog |
| Shroom3 | shroom |
| Siae | yolk sac gene 2 |
| Sigirr | single Ig IL-1 receptor related protein |
| Slc15a2 | solute carrier family 15 (H+/peptide transporter), member 2 |
| Slc22a7 | Mus musculus, Similar to solute carrier family 22 (organic anion transporter) |
| Slc23a2 | solute carrier family 23 (nucleobase transporters), member 2 |
| Slc25a11 | solute carrier family 25 (mitochondrial carrier; oxoglutarate carrier), member 11 |
| Slc25a13 | solute carrier family 25 (mitochondrial carrier; adenine nucleotide translocator), member 13 |
| Slc25a19 | solute carrier family 25 (mitochondrial deoxynucleotide carrier), member 19 |
| Slc25a5 | solute carrier family 25 (mitochondrial carrier; adenine nucleotide translocator), member 5 |
| Slc28a2 | solute carrier family 28 (sodium-coupled nucleoside transporter), member 2 |
| Slc29a2 | solute carrier family 29 (nucleoside transporters), member 2 |
| Slc2a1 | solute carrier family 2 (facilitated glucose transporter), member 1 |
| Slc31a1 | expressed sequence AI787263 |
| Slc31a2 | solute carrier family 31, member 2 |
| Slc34a2 | solute carrier family 34 (sodium phosphate), member 2 |
| Slc35a1 | solute carrier family 35 (CMP-sialic acid transporter), member 1 |
| Slc39a14 | solute carrier family 39 (zinc transporter), member 14 |
| Slc6a13 | Mus musculus, Similar to solute carrier family 6 (neurotransmitter transporter, GABA) |
| Slc7a6 | solute carrier family 7 (cationic amino acid transporter, y+ system), member 6 |
| Slc9a3r1 | solute carrier family 9 (sodium/hydrogen exchanger), isoform 3 regulator 1 |
| Slu7 | SLU7 splicing factor homolog |
| Smad5 | MAD homolog 5 |
| Smarcd1 | SWI/SNF related, matrix associated, actin dependent regulator of chromatin |
| Smtn | smoothelin |
| Snap23 | synaptosomal-associated protein, 23kD |
| Snrpb2 | U2 small nuclear ribonucleoprotein B |
| Snrpe | small nuclear ribonucleoprotein E |
| Snx5 | sorting nexin 5 |
| Soat1 | sterol O-acyltransferase 1 |
| Socs3 | cytokine inducible SH2-containing protein 3 |
| Sp1 | trans-acting transcription factor 1 |
| Sparc | secreted acidic cysteine rich glycoprotein |
| Spata2 | spermatogenesis associated 2 |
| Spfh2 | SPFH domain family, member 2 |
| Spin | spindlin |
| Srp14 | signal recognition particle 14 kDa (homologous Alu RNA binding protein) |
| Srrm2 | serine/arginine repetitive matrix 2 |
| St7 | suppression of tumorigenicity 7 |
| Stk11 | serine/threonine kinase 11 |
| Stk25 | serine/threonine kinase 25 (yeast) |
| Stk3 | serine/threonine kinase 3 |
| Stx1bl | expressed sequence AU015105 |
| Sucla2 | succinate-Coenzyme A ligase, ADP-forming, beta subunit |
| Sui1-rs1 | suppressor of initiator codon mutations, related sequence 1 |
| Syt4 | synaptotagmin 4 |
| Tacstd1 | tumor-associated calcium signal transducer 1 |
| Taf12 | TAF12 RNA polymerase II, TATA box binding protein (TBP)-associated factor, 20 kDa |
| Tbl3 | transducin (beta)-like 3 |
| Tbx19 | T-box 19 |
| Tcf20 | transcription factor 20 |
| Tcfe2a | transcription factor E2a |
| Tcp1 | t-complex protein 1 |
| Tera | teratocarcinoma expressed, serine rich |
| Tex101 | testis expressed gene 101 |
| Tgfb1i4 | transforming growth factor beta 1 induced transcript 4 |
| Thap11 | hypothetical protein, MNCb-2032 |
| Thap2 | THAP domain containing, apoptosis associated protein 2 |
| Tia1 | cytotoxic granule-associated RNA binding protein 1 |
| Timm10 | Mus musculus, translocase of inner mitochondrial membrane 10 homolog |
| Timm8b | translocase of inner mitochondrial membrane 8 homolog b (yeast) |
| Tirap | toll-interleukin 1 receptor (TIR) domain-containing adaptor protein |
| Tlk2 | tousled-like kinase 2 (Arabidopsis) |
| Tmc6 | transmembrane channel-like gene family 6 |
| Tmem2 | transmembrane protein 2 |
| Tmem48 | transmembrane protein 48 |
| Tmsb10 | thymosin, beta 10 |
| Tnfaip1 | tumor necrosis factor, alpha-induced protein 1 (endothelial) |
| Tnfaip2 | tumor necrosis factor, alpha-induced protein 2 |
| Tnk2 | tyrosine kinase, non-receptor, 2 |
| Tollip | toll interacting protein |
| Tpm2 | tropomyosin 2, beta |
| Trfp | Trf (TATA binding protein-related factor)-proximal protein homolog |
| Trh | thyrotropin releasing hormone |
| Trim46 | expressed sequence AI385631 |
| Trio | triple functional domain (PTPRF interacting) |
| Trip12 | thyroid hormone receptor interactor 12 |
| Tro | trophinin |
| Tspan6 | transmembrane 4 superfamily member 6 |
| Ttc3 | tetratricopeptide repeat domain |
| Tubb3 | tubulin, beta 3 |
| Tubb5 | tubulin, beta 5 |
| Txndc4 | endoplasmic reticulum resident protein 44kDa |
| Txnl | thioredoxin-like (32kD) |
| Ube2d2 | ubiquitin-conjugating enzyme E2D 2 |
| Ube2l3 | ubiquitin-conjugating enzyme E2L 3 |
| Ube2n | ubiquitin-conjugating enzyme E2N |
| Ube2v2 | ubiquitin-conjugating enzyme E2 variant 2 |
| Ube3a | ubiquitin protein ligase E3A |
| Ublcp1 | ubiquitin-like domain containing CTD phosphatase 1 |
| Ugalt2 | UDP-galactose translocator 2 |
| Uhrf1 | nuclear protein 95 |
| Unc5c | unc5 homolog 3 |
| Upf3b | UPF3 regulator of nonsense transcripts homolog B |
| Uqcrc1 | ubiquinol-cytochrome c reductase core protein 1 |
| Usf2 | upstream transcription factor 2 |
| Usp1 | Mus musculus, Similar to ubiquitin specific protease 1 |
| Usp2 | ubiquitin specific protease 2 |
| Usp21 | ubiquitin specific protease 21 |
| Usp25 | ubiquitin specific protease 25 |
| Usp3 | Mus musculus, Similar to ubiquitin specific protease 3 |
| Usp47 | aryl-hydrocarbon receptor |
| Vcp | valosin containing protein |
| Vprbp | Vpr (HIV-1) binding protein |
| Vps25 | vacuolar protein sorting 25 |
| Vta1 | Vps20-associated 1 homolog |
| Wdr1 | WD repeat domain 1 |
| Wdr42a | WD repeat domain 42A |
| Wwc1 | Mus musculus similar to BAI1-associated protein 1 |
| Xpnpep1 | cytosolic aminopeptidase P |
| Xrcc1 | X-ray repair complementing defective repair in Chinese hamster cells 1 |
| Ylpm1 | ZAP3 protein |
| Ywhab | Mus musculus tyrosine 3-monooxygenase/tryptophan 5-monooxygenase activation protein |
| Zbtb10 | zinc finger and BTB domain containing 10 |
| Zbtb16 | zinc finger and BTB domain containing 16 |
| Zc3h11a | Mus musculus, Similar to KIAA0663 gene product |
| Zfp276 | zinc finger protein (C2H2 type) 276 |
| Zfp688 | Mus musculus similar to Zinc finger |
| Zfr | zinc finger RNA binding protein |
| Zp2 | zona pellucida glycoprotein 2 |
