# Supplementary material for: Conserved and Differential Effects of Dietary Energy Intake on the Hippocampal Transcriptomes of Females and Males
Source: PLoS One. 2008 Jun 11;3(6):e2398. doi: 10.1371/journal.pone.0002398 (PMC2405949; doi:10.1371/journal.pone.0002398)
Supplement: Table S2 — Description of the gene pathways significantly altered in figures 13 and 14. (0.10 MB DOC) [file pone.0002398.s002.doc]

**Supplementary Table 2: Description of the gene pathways significantly altered in figures 13 and 14.**

| **1.** Muscle_Myosin | Genes related to muscle myosin |
| --- | --- |
| **2.** GLUCO | Genes involved in glucose processing |
| **3.** Glycolysis_and_Gluconeogenesis | Genes involved in glycolysis and gluconeogenesis |
| **4.** Bile_acid_biosynthesis | Genes involved in bile acid biosynthesis |
| **5.** ROS | Reactive oxidative species related genes |
| **6.** Cdc25 | The protein phosphatase Cdc25 is phosphorylated by Chk1 and activates Cdc2 to stimulate eukaryotic cells into M phase. |
| **7.** GlycolysisPathway | Glycolysis is an evolutionarily conserved pathway by which one glucose molecule is converted to two pyruvate molecules for a gain of 2 ATP. |
| **8.** Nos1 | Glutamate stimulates NMDA-mediated calcium influx, which promotes nitric oxide synthesis from arginine by neuronal nitric oxide synthase, activating guanylate cyclase. |
| **9.** Eif2 | Eukaryotic initiation factor 2 (EIF2) initiates translation by transferring Met-tRNA to the 40S ribosome in a GTP-dependent process. |
| **10.** FOSB | FOSB gene expression and drug abuse |
| **11.** Mef2d | Mef2 transcription factors promote calcium-induced apoptosis in T cells and are regulated by MAP kinases and histone deacetylases. |
| **12.** Ndk_Dynamin | Synaptic vesicle endocytosis is coordinated by dephosphins, which are coordinately dephosphorylated on calmodulin- and calcineurin-modulated calcium influx. |
| **13.** Androgen_genes | Genes annotated in NetAffx as androgen related |
| **14.** Peng_GLUT_UP | Genes upregulated in response to glutamine starvation |
| **15.** Glycolysis | Glycolysis related genes |
| **16.** Glycogen_Metabolism | Genes involved in glycogen metabolism |
| **17.** Peng_Leucine_UP | Genes upregulated in response to leucine starvation |
| **18.** Carbon_Fixation | Genes related to carbon fixation in photosynthetic organisms |
| **19.** ST_Interferon_Gamma | The interferon gamma pathway resembles the JAK-STAT pathway and activates STAT transcription factors. |
| **20.** ST_Type_I_Interferon | Type I interferon is an antiviral cytokine that induces a JAK-STAT type pathway leading to ISGF3 activation and a cellular antiviral response. |
| **21.** TCA | Tricarboxylic acid related genes |
| **22.** Arf | Cyclin-dependent kinase inhibitor 2A is a tumor suppressor that induces G1 arrest and can activate the p53 pathway, leading to G2/M arrest. |
| **23.** Cbl | Activated EGF receptors undergo endocytosis into clathrin-coated vesicles, where they are recycled to the membrane or ubiquitinated by Cbl. |
| **24.** Ctla4 | T cell activation requires interaction with an antigen-MHC-I complex on an antigen-presenting cell (APC), as well as CD28 interaction with the APC's CD80 or 86. |
| **25.** Fxr | The nuclear receptor transcription factors FXR and LXR are activated by cholesterol metabolites and regulate cholesterol homeostasis. |
| **26.** Ifna | Interferon alpha, active in the immune response, binds to the IFN receptor and activates Jak1 and Tyk2, which phosphorylate Stat1 and Stat2. |
| **27.** Intrinsic | The intrinsic prothrombin activation pathway is activated by traumatized blood vessels and induces clot formation. |
| **28.** Leptin | Leptin is a peptide secreted by adipose tissue that, in skeletal muscle, promotes fatty acid oxidation, decreases cells' lipid content, and promotes insulin sensitivity. |
| **29.** Pgc1a | PCG-1a is expressed in skeletal muscle, heart muscle, and brown fat, and is a coactivator for receptors such as glucocorticoid receptor and thyroid hormone receptor. |
| **30.** Rarrxr | RXR and RAR suppress transcription in the absence of ligand and, on binding trans- or 9-cis-retinoic acid, are ubiquitinated to allow transcription to proceed. |
| **31.** Calcineurin | Increased intracellular calcium activates the phosphatase calcineurin in differentiating keratinocytes. |
| **32.** Caspase | Caspases are cysteine proteases active in apoptosis; caspase-8 and 9 cleave and activate other caspases, while 3, 6, and 7 cleave cellular targets. |
| **33.** Sodd | Some members of the tumor necrosis factor receptor family have cytoplasmic death domains that promote apoptosis when active and are repressed by silencers called SODDs. |
| **34.** Keratinocyte | Keratinocyte differentiation, which models the differentiation of epidermal cells, requires the four main MAP kinase pathways. |
| **35.** VIP | Apoptosis of activated T cells is inhibited by vasoactive intestinal peptide (VIP) and its relative PACAP. |
| **36.** Proteasome | Ubiquitinated proteins are targeted for proteolytic degradation by the proteasome, where they are unfolded and degraded to small peptides in an ATP-dependent process. |
| **37.** Akap13 | A-kinase anchor protein 13 (AKAP13) localizes protein kinase A holoenzyme and is a nucleotide exchange factor for Rho/Rac. |
| **38.** GPCRs_Class_C_Metabotropic_  Glutamate_Pheromone | The group of G-protein coupled receptors that are structurally/functionally related to the metabotropic glutamate receptors |
| **39.** Par1 | Activated extracellular thrombin cleaves and activates the G-protein coupled receptors PAR1 and PAR4, which activate platelets. |
| **40.** Pentose_Phosphate | Pentose Phosphate Pathway |
| **41.** Glycolysis | Glycolysis is an evolutionarily conserved pathway by which one glucose molecule is converted to two pyruvate molecules for a gain of 2 ATP. |
| **42.** Mef2d | Mef2 transcription factors promote calcium-induced apoptosis in T cells and are regulated by MAP kinases and histone deacetylases. |
| **43.** Eryth | Erythropoietin selectively stimulates erythrocyte differentiation from CFU-GEMM cells in bone marrow. |
| **44.** Prostaglandin_and_Leukotriene_  Metabolism | Genes related to prostaglandin and leukotriene metabolism |
| **45.** Androgen_genes | Genes annotated in NetAffx as androgen related |
| **46.** Cell_Adhesion_Molecule_  Activity | Obsolete by GO - mediates the adhesion of the cell to other cells or to the extracellular matrix. |
| **47.** Nos1 | Glutamate stimulates NMDA-mediates calcium influx, which promotes nitric oxide synthesis from arginine by neuronal nitric oxide synthase, activating guanylate cyclase. |
| **48.** GABA | Gamma-aminobutyric acid (GABA) is an inhibitory neurotransmitter whose receptor is regulated by Plic-1, gephyrin, and GABARAP, which promote receptor clustering. |
| **49.** Inflam | Interleukins and TNF serve as signals to coordinate the inflammatory response, in which macrophages recruit and activate neutrophils, fibroblasts, and T cells. |
| **50.** Reductive_Carboxylate_Cycle_  CO2_Fixation | Reductive carboxylate cycle CO_2_ fixation related genes |
| **51.** Proteasome | Ubiquitinated proteins are targeted for proteolytic degradation by the proteasome, where they are unfolded and degraded to small peptides in an ATP-dependent process. |
| **52.** p35_Alzheimers | p35, a neuron-specific activator of cyclin-dependent kinase 5, is cleaved to p25 in Alzheimer's disease and promotes hyperphosphorylated tau formation and apoptosis. |
| **53.** GLUCO | Genes involved in glucose processing |
| **54.** FBW7 | Cyclin E interacts with cell cycle checkpoint kinase cdk2 to allow transcription of genes required for S phase, including transcription of additional cyclin E. |
| **55.** Arginine_and_Proline_  Metabolism | Genes involved in arginie and proline metabolism |
| **56.** Tert | hTERC, the RNA subunit of telomerase, and hTERT, the catalytic protein subunit, are required for telomerase activity and are overexpressed in many cancers. |
| **57.** Eif2 | Eukaryotic initiation factor 2 (EIF2) initiates translation by transferring Met-tRNA to the 40S ribosome in a GTP-dependent process. |
| **58.** IL12 | IL12 and Stat4 Dependent Signaling Pathway in Th1 Development |
| **59.** Pgc1a | PCG-1a is expressed in skeletal muscle, heart muscle, and brown fat, and is a coactivator for receptors such as glucocorticoid receptor and thyroid hormone receptor. |
| **60.** Human_CD34_Enriched_  Transcription_Factors | Human CD34 enriched transcription factors related genes |
| **61.** HDAC | Myocyte enhancer factor MEF2 activates transcription of genes required for muscle cell differentiation and is inhibited by histone deacetylases. |
| **62.** Glycolosis_and_Gluconeogenesis | Genes involved in glycolysis and gluconeogenesis |
| **63.** G2 | Activated Cdc2-cyclin B kinase regulates the G2/M transition; DNA damage stimulates the DNA-PK/ATM/ATR kinases, which inactivate Cdc2. |
| **64.** PS1 | Presenilin is required for gamma-secretase activity to activate Notch signaling; presenilin also inhibits beta-catenin in the Wnt/Frizzled pathway. |
| **65.** CR CAM | PNAS 2002: Cancer related genes involved in cell adhesion and metalloproteinases |
| **66.** Spry | Four members of the Sprouty protein family block proliferative EGF signals by binding Grb-2, preventing Ras and MAP kinase activation. |
| **67.** Mitr | The MyoD/MEF2 transcription factors induce muscle cell differentiation and are repressed by the transcriptional repressor MITR. |
| **68.** Ctla4 | T cell activation requires interaction with an antigen-MHC-I complex on an antigen-presenting cell (APC), as well as CD28 interaction with the APC's CD80 or 86. |
| **69.** Bile_acid_biosynthesis | Genes involved in bile acid biosynthesis |
| **70.** MalateX | The tricarboxylate transfer pathway shuttles acetyl groups of acetyl-CoA between mitochondria and the cytoplasm. |
| **71.** Ndk_Dynamin | Synaptic vesicle endocytosis is coordinated by dephosphins, which are coordinately dephosphorylated on calmodulin- and calcineurin-modulated calcium influx. |
| **72.** p53 | p53 induces cell cycle arrest or apoptosis under conditions of DNA damage. |
| **73.** Type_III_secretion_system | Type III secretion system related genes |
| **74.** ATP_synthesis | Genes involved in ATP synthesis |
| **75.** ROS | Reactive oxidative species related genes |
| **76.** TCA | Tricarboxylic acid related genes |
| **77.** VIP | Apoptosis of activated T cells is inhibited by vasoactive intestinal peptide (VIP) and its relative PACAP. |
| **78.** Cdc25 | The protein phosphatase Cdc25 is phosphorylated by Chk1 and activates Cdc2 to stimulate eukaryotic cells into M phase. |
| **79.** RB | The ATM protein kinase recognizes DNA damage and blocks cell cycle progression by phosphorylating chk1 and p53, which normally inhibits Rb to allow G1/S transitions. |
| **80.** Arf | Cyclin-dependent kinase inhibitor 2A is a tumor suppressor that induces G1 arrest and can activate the p53 pathway, leading to G2/M arrest. |
